# Fetuin-A and its genetic association with cardiometabolic disease

**DOI:** 10.1038/s41598-023-48600-9

**Published:** 2023-12-06

**Authors:** Lawien Al Ali, Yordi J. van de Vegte, M. Abdullah Said, Hilde E. Groot, Tom Hendriks, Ming Wai Yeung, Erik Lipsic, Pim van der Harst

**Affiliations:** 1 Department of Cardiology, University of Groningen, University Medical Center Groningen, Hanzeplein 1, PO Box 30.001, 9700 RB Groningen, the Netherlands; 2 Department of Heart and Lungs, University of Utrecht, University Medical Center Utrecht, Utrecht, the Netherlands

**Keywords:** Cardiology, Calcification, Cardiovascular genetics, Epidemiology, Genetics research

## Abstract

Fetuin-A acts as both an inhibitor of calcification and insulin signaling. Previous studies reported conflicting results on the association between fetuin-A and cardiometabolic diseases. We aim to provide further insights into the association between genetically predicted levels of fetuin-A and cardiometabolic diseases using a Mendelian randomization strategy. Genetic variants associated with fetuin-A and their effect sizes were obtained from previous genetic studies. A series of two-sample Mendelian randomization analyses in 412,444 unrelated individuals from the UK Biobank did not show evidence for an association of genetically predicted fetuin-A with any stroke, ischemic stroke, or myocardial infarction. We do find that increased levels of genetically predicted fetuin-A are associated with increased risk of type 2 diabetes (OR = 1.21, 95%CI 1.13–1.30, *P* =  < 0.01). Furthermore, genetically predicted fetuin-A increases the risk of coronary artery disease in individuals with type 2 diabetes, but we did not find evidence for an association between genetically predicted fetuin-A and coronary artery disease in those without type 2 diabetes (*P* for interaction = 0.03). One SD increase in genetically predicted fetuin-A decreases risk of myocardial infarction in women, but we do not find evidence for an association between genetically predicted fetuin-A and myocardial infarction in men (*P* for interaction =  < 0.01). Genetically predicted fetuin-A is associated with type 2 diabetes. Furthermore, type 2 diabetes status modifies the association of genetically predicted fetuin-A with coronary artery disease, indicating that fetuin-A increases risk in individuals with type 2 diabetes. Finally, higher genetically predicted fetuin-A reduces the risk of myocardial infarction in women, but we do not find evidence for an association between genetically predicted fetuin-A and myocardial infarction in men.

## Introduction

Fetuin-A, also known as α-Heremans-Schmid glycoprotein (AHSG), is a liver-synthesized protein that acts as a systemic inhibitor of ectopic calcification^1^. This protein can additionally inhibit insulin signaling at tyrosine kinase level^2^ and is associated with insulin resistance^3^.

Several prospective population-based studies investigated the association between circulating fetuin-A levels and risk of cardiovascular disease (CVD) and/or type 2 diabetes but have reported conflicting results^4–7^. For example, the European Prospective Investigation into Cancer and Nutrition (EPIC)-Potsdam Study found higher plasma fetuin-A to be associated with an increased risk of myocardial infarction (MI), ischemic stroke (IS)^4^, and type 2 diabetes mellitus^5^. In the Rancho Bernardo study, however, higher fetuin-A levels were associated with lower risk of CVD mortality in adults without diabetes but higher risk of CVD mortality in those with diabetes^6^. Similar results were reported for the Cardiovascular Health Study (CHS). Higher fetuin-A levels were associated with lower CVD risk in individuals without type 2 diabetes, but a trend in the opposite direction was observed among individuals with type 2 diabetes^7^.

Several studies have investigated the genetic association between fetuin-A plasma levels and CVD utilizing Mendelian randomization methods^8–10^. Mendelian randomization is a method that, under specific assumptions, intends to estimate generally unconfounded effects. One such study observed that increased levels of genetically predicted fetuin-A increase the risk of MI^8^, while another study did not find evidence of an association with CVD^9^. In contrast to traditional observational studies, a Mendelian randomization study did not find evidence supporting an association between fetuin-A and type 2 diabetes^10^. Since the publication of these previous Mendelian randomization studies, novel genetic variants associated with fetuin-A have been identified in a genome wide association study^11^. In addition, new advances have been made in Mendelian randomization strategies, which can better detect and take into account potential biases. Furthermore, the rise of large biobanks, combining a broad array of phenotypic and genetic data, allows for the assessment of a comorbidity dependent effect of fetuin-A on CVD.

The first objective of this study is to assess the association of genetically predicted fetuin-A with type 2 diabetes and cardiovascular outcomes, including coronary artery disease, myocardial infarction, any stroke, and ischemic stroke. The second objective is to assess whether a comorbidity dependent effect exists between genetically predicted fetuin-A and cardiovascular outcomes.

## Results

### Baseline characteristics

A total of 412,444 unrelated individuals from the general population were included in the current study, after exclusion of 14,242 individuals with missing information on covariates, 1341 individuals who failed genetic quality control, and 74,467 individuals based on genetic relatedness. The mean age at inclusion was 57.0 ± 8.1 years and the population consisted of 46.1% men. The median follow-up was 11.7 years (IQR 10.9–12.4). The mean body mass index (BMI) was 27.4 ± 4.8 kg/m^2^. The combined incidence and prevalence of coronary artery disease, myocardial infarction, any stroke, ischemic stroke and type 2 diabetes were 10.1%, 5.1%, 5.0%, 3.0% and 7.0%, respectively. Additional information on the cohort is provided in Table 1. Single SNP exposure, outcome and exposure-outcome associations, including F-statistics, data harmonization, Steiger filtering, and Wald estimates for all outcomes, can be found in Online Table 1. F-statistics were obtained from the exposure sample and not in the outcome sample, as we did not have direct measurements of plasma fetuin-A in the UK Biobank. The exposure and outcome samples did not overlap in individuals. An overview of the genetic variant selection method for the main and sensitivity analysis can be found in Supplementary Fig. 1.

**Table 1:** Baseline characteristics.

	Total sample (n = 412,444)	Coronary artery disease (n cases = 41,550)	Myocardial infarction (n cases = 20,864)	Any stroke (n cases = 20,632)	Ischemic stroke (n cases = 12,298)	Type 2 diabetes (n cases = 28,333)
Age, y	57.0 ± 8.1	61.6 ± 8.1	61.5 ± 6.7	61.6 ± 6.7	62.0 ± 6.5	60.2 ± 7.1
Sex, male %	46.1	69.4	74.4	57.2	59.5	60.6
Ethnicity, %						
White	93.7	93.9	93.2	94.3	94.9	86.1
Black	1.7	1.2	1.0	1.7	1.5	3.6
Asian	2.5	3.6	3.7	2.2	2.0	6.8
Mixed	0.6	0.5	1.0	0.5	0.4	0.7
Unknown	1.5	1.5	1.5	1.3	1.1	2.7
BMI, kg/m^2^	27.4 ± 4.8	29.0 ± 5.0	29.0 ± 4.9	28.4 ± 5.1	28.4 ± 5.0	31.6 ± 5.7
Hyperlipidemia, %	19.0	56.2	61.3	32.4	33.3	38.5
Systolic blood pressure, mmHg	133.2 ± 18.0	138.3 ± 18.0	137.9 ± 18.2	138.4 ± 18.6	139 ± 18.9	138.3 ± 17.1
Diastolic blood pressure, mmHg	82.1 ± 8.6	82.3 ± 8.9	82.1 ± 9.0	82.8 ± 9.0	83.0 ± 9.1	83.1 ± 8.5
Hypertension, %	36.0	46.0	44.8	46.6	48.6	46.6
Smoking behavior, %						
Never or < 100 cigarettes	56.7	42.3	38.9	46.0	46.0	45.9
Stopped < = 12 months	0.5	0.7	0.8	0.7	0.6	0.7
Stopped > 12 months	32.0	42.7	43.8	38.2	38.7	40.1
Active smoking occasionally	2.8	2.9	3.3	2.9	2.8	2.8
Active smoking daily	7.9	11.4	13.2	12.3	11.8	10.4
Family history of cardiac disease	43.8	56.9	57.8	49.0	50.2	48.5
Coronary artery disease, %	10.1	100.0	100.0	28.6	30.6	29.7
Myocardial infarction, %	5.1	50.2	100.0	15.6	16.6	15.8
Stroke, %	5.0	14.2	15.4	100.0	100.0	12.1
Ischemic stroke, %	3.0	9.0	9.8	59.6	100.0	7.5
Type 2 diabetes, %	7.0	21.3	22.8	17.4	18.2	100.0

Baseline characteristics of up to 422,444 individuals in the UK Biobank for the total sample and per disease status. Disease status includes prevalence and incidence. Type 2 diabetes status was only present in 403,289 individuals. Continuous and normally distributed variables are presented as mean ± SD, binary variables as percentages.

### Mendelian randomization analyses

**Figure 1:**
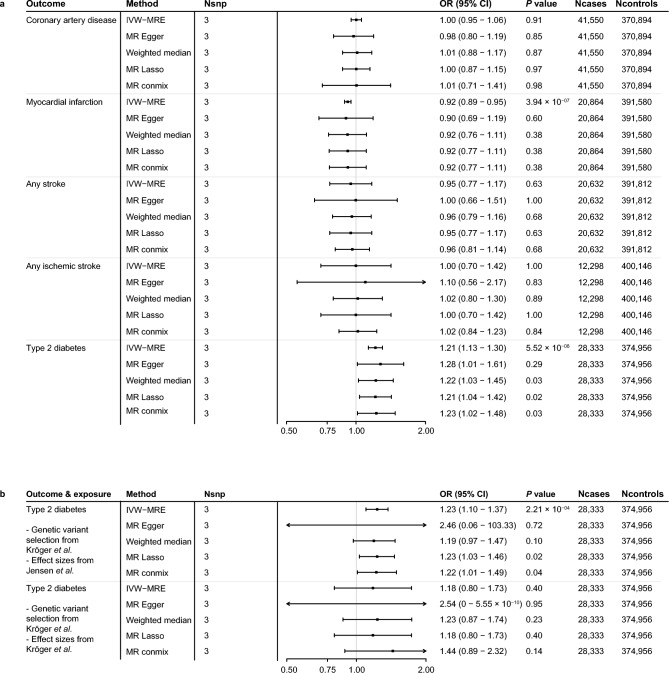
Two sample Mendelian randomization analysis of fetuin-A with coronary artery disease, myocardial infarction, stroke, ischemic stroke, and type 2 diabetes. (**a**) Forest plot of the results of the univariable two-sample Mendelian randomization analyses of fetuin-A on CVDs and type 2 diabetes in the UK Biobank. Weak-instrument bias was assessed by calculating the F-statistic using the following formula: *F* = *R*^2^(*n −* 2)/(1* − R*^2^)*.* Here, *n* is the sample size used to obtain the fetuin-A estimates and R^2^ is the amount of variance in fetuin-A explained by the genetic variant^24^. An F-statistic < 10 was considered to indicate weak-instrument bias. MR-Steiger filtering was performed to assess reversed causation. MR estimates were obtained using an inverse variance weighted multiplicative random effects model (IVW-MRE), MR-Egger model, MR Lasso method, weighted median approach, and using the contamination mixture model. Sensitivity analyses not shown in the current article showed no evidence for weak instrument bias and genetic variants displaying reversed causation were filtered. Neither did we find evidence for unbalanced horizontal pleiotropy as indicated by the Rucker framework, the MR-Egger test was therefore not taken forward as primary analysis. Genetic effect estimates are reported in odds ratios per one SD increase in genetically determined fetuin-A. (**b**) Sensitivity two-sample Mendelian randomization analyses between fetuin-A and type 2 diabetes in the UK Biobank. The results show that alteration of the SNP selection, using the three genetic variants used in the study from Kroger et al*.* with the exposure effect size from the GWAS from Jensen et al., does not change the significant genetic association between fetuin-A and type 2 diabetes. Alteration of the effect size of these variants, using the three genetic variants used in the study from Kroger et al*.* with the exposure effect sizes obtained from Kroger et al*.*, does negate the significant association between fetuin-A and type 2 diabetes. Instrument strength therefore seems the key difference in the discrepancy of results between the current study and the previous research from Kröger et al. Genetic effect estimates are reported in odds ratios per one SD increase in genetically determined fetuin-A. *MR* Mendelian randomization; *MR* Mendelian randomization; *IVW-MRE* Inverse variance weighted multiplicative random effects; *commix* Contamination mixture model; *SNP* Single nucleotide polymorphism, *OR* Odds ratio; *CI* Confidence interval.

We performed a series of two-sample MR analyses to assess whether genetically predicted fetuin-A is associated with cardiovascular traits and type 2 diabetes. Scatterplots, forest plots, funnel plots and leave-one-out plots of the two-sample MR analyses are provided in Supplementary Figs. 2, 3, 4 and 5.We did not find evidence for an association between genetically predicted fetuin-A and coronary artery disease (OR = 1.00, 95% CI 0.95–1.06, *P* = 0.91) in the combined cohort of individuals with and without type 2 diabetes. One SD increase of genetically predicted fetuin-A was associated with decreased risk of myocardial infarction in the main IVW-MRE analysis (OR = 0.92, 95% CI 0.89–0.95, *P* =  < 0.01), but this association could not be substantiated in the MR sensitivity analyses (Fig. 1a, Online Table 2). We did not find evidence for an association between genetically predicted fetuin-A and any stroke (OR = 0.95, 95% CI 0.77–1.17, *P* = 0.63), and ischemic stroke (OR = 1.00, 95% CI 0.70–1.42, *P* = 0.99) in the main IVW-MRE analyses (Fig. 1a). However, we did find that one SD increase in genetically predicted fetuin-A increased risk of type 2 diabetes (OR = 1.21, 95% CI 1.13–1.30, *P* =  < 0.01), which was further supported by sensitivity analyses (MR Lasso OR = 1.21, 95% CI 1.04–1.42, *P* = 0.02, weighted median OR = 1.22, 95% CI 1.03–1.45, *P* = 0.03, and MR contamination mixture model OR = 1.23, 95% CI 1.02–1.48, *P* = 0.04). In the leave-one-out analysis, we find that the effect of genetically predicted fetuin-A on type 2 diabetes is no longer significant if SNP rs11017848 is excluded (OR = 1.178, 95%CI = 0.893–1.554, *P* = 0.247; Online Table 3 and Supplementary Fig. 2). Visual inspection of the funnel plot in Supplementary Fig. 2 shows that rs11017848 is an unlikely outlier in the association between genetically predicted fetuin-A and type 2 diabetes. Using the Rücker framework, we did not find evidence for unbalanced horizontal pleiotropy in these MR estimates (Online Table 3).

We repeated our analysis using (a) a subset of 11 independent genetic variants at a more lenient clumping threshold of R^2^ = 0.05 (rs4917, rs9290835, rs4488820, rs13073740, rs4686799, rs843991, rs11918289, rs745588, rs11017848, rs4615068, and rs6809265), (b) a subset of variants used in the study of Kröger et al.^10^ (rs4917, rs2070633, rs2248690), and (c) a subset of genetic variants used in the study from Fisher et al.^8^ (rs4917, rs2070633, rs2248690, and rs2070635). These results were consistent with our findings (Online Tables 1, 2, 3 and 4).

We performed additional sensitivity analyses in order to compare current MR results with previous studies. The MR analyses between genetically predicted fetuin-A and type 2 diabetes were repeated using the subset of variants used in the study of Kröger et al.^10^, using the effect sizes of from the Potsdam part of the EPIC-InterAct study instead of the effect sizes obtained from the meta-analysis of the CHARGE consortium^11^. We find that using the effect sizes of from the Potsdam part of the EPIC-InterAct study nullifies the association between fetuin-A and type 2 diabetes (OR = 1.30, 95% CI 0.87–1.94, *P* = 0.20; Supplementary Table 1, Fig. 1b).

### Interaction between fetuin-A and type 2 diabetes

**Figure 2:**
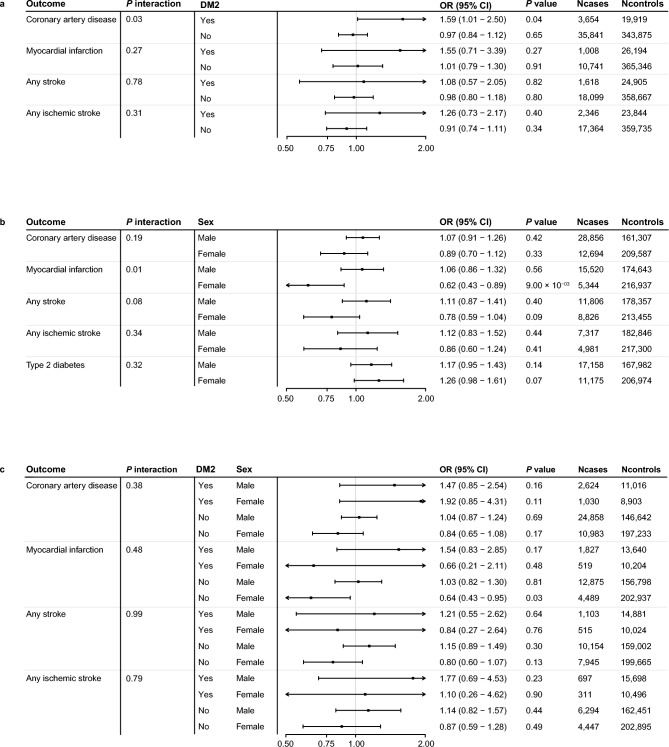
Genetic interaction analysis of fetuin-A with coronary artery disease, myocardial infarction, stroke, ischemic stroke, and type 2 diabetes. (**a**) Two-way interaction between genetic risk score of fetuin-A and phenotypical type 2 diabetes in the association with coronary artery disease, myocardial infarction, stroke and ischemic stroke. The genetic risk scores were constructed based on all 3 3 genetic variants that were found independently associated with fetuin-A after clumping (R^2^ < 0.01 within a 2.5 Mb window on either site). The genetic risk scores was constructed by summing the number of alleles (0, 1 or 2) for each individual after multiplication with the effect size between the SNP and fetuin-A. Type 2 diabetes had to be diagnosed before any of the assessed health outcomes to be counted as previously diagnosed. Logistic regression analysis were performed using an interaction term between fetuin-A and type 2 diabetes. Genetic effect estimates are reported in odds ratios per one SD increase in genetically determined fetuin-A. (**b**) Two-way interaction between genetic risk score of fetuin-A and sex in the association with coronary artery disease, myocardial infarction, stroke and ischemic stroke. Methodology as described in figure Section 1B) Genetic effect estimates are reported in odds ratios per one SD increase in genetically determined fetuin-A. (**c**) Three-way interaction between genetic risk score of fetuin-A, sex, and type 2 diabetes in the association with coronary artery disease, myocardial infarction, stroke and ischemic stroke. Methodology as described in figure Section 1B) Genetic effect estimates are reported in odds ratios per one SD increase in genetically determined fetuin-A. *SNP* Single nucleotide polymorphism, *OR* Odds ratio; *CI* Confidence interval.

We then sought out to investigate a potential interaction between fetuin-A and type 2 diabetes on cardiovascular outcomes (Fig. 2a). We find that genetically predicted fetuin-A increases the risk of coronary artery disease in individuals with type 2 diabetes (OR = 1.59, 95% CI 1.01–2.50, *P* = 0.04), but we do not find such an association between genetically predicted fetuin-A and coronary artery disease in individuals without type 2 diabetes (OR = 0.97, 95% CI 0.84–1.12, *P* = 0.65; *P* for interaction = 0.03). This significant interaction was not replicated using the other genetic risk scores that were constructed for sensitivity purposes (Online Table 5). We did not find evidence for an interaction between genetically predicted fetuin-A and type 2 diabetes in the association with myocardial infarction, any stroke, and ischemic stroke (Online Table 5).

### Interaction between fetuin-A and additional risk factors for CVD

We find that genetically predicted fetuin-A reduces the risk of myocardial infarction in women (OR = 0.62, 95% CI 0.43–0.89, P =  < 0.01), but we do not find evidence for an association between genetically predicted fetuin-A and myocardial infarction in men (OR = 1.06, 95% CI 0.86–1.32, *P* = 0.56; *P* for interaction =  < 0.01; Fig. 2b, Online Table 7). We do not find evidence for an interaction between genetically predicted fetuin-A and sex in the association with coronary artery disease, any stroke or ischemic stroke (Fig. 2b, Online Table 6). We also do not find evidence for an interaction of genetically predicted fetuin-A with age, BMI, hypertension, or hyperlipidemia in the association with coronary artery disease, myocardial infarction, any stroke, ischemic stroke, or type 2 diabetes. In addition, we did not find evidence for a three-way interaction of type 2 diabetes and sex in the association between genetically predicted fetuin-A and coronary artery disease, myocardial infarction, any stroke, and ischemic stroke (Fig. 2c, Online Table 7).

## Discussion

In this large-scale prospective population-based cohort study, we present evidence that higher genetically determined fetuin-A increases risk of type 2 diabetes development. Furthermore, we provide evidence that type 2 diabetes status modifies the association of genetically predicted fetuin-A with coronary artery disease. In addition, we are the first to show that genetically predicted fetuin-A decreases the risk of myocardial infarction in women, but we do not find evidence for an association between genetically predicted fetuin-A and myocardial infarction in men. We do not find evidence for an association of genetically predicted fetuin-A with stroke.

### Comparison with previous research

In contrast to a previous Mendelian randomization study^10^, we find consistent evidence for an association between genetically predicted fetuin-A and type 2 diabetes in both our main and most of our sensitivity analyses. One possible explanation of the discrepancy of these results is a larger strength of the genetic instruments in the current study, as we find that using the effect sizes of the genetic variants on fetuin-A obtained from the Potsdam part of the EPIC-InterAct study^10^ instead of those from the CHARGE meta-analysis^11^ nullifies the association between fetuin-A and type 2 diabetes. Both cohorts have equal amount of type 2 diabetes cases, which makes it unlikely that this factor contributes to the discrepancy of the results. Population stratification could potentially contribute to the discrepancy of results, although both outcome cohorts included mostly, but not solely, individuals of European descent. We also note that that the results of our main analysis between genetically predicted fetuin-A and type 2 diabetes were no longer significant after exclusion of rs11017848, a genetic variant that was not included in previous Mendelian randomization studies. We therefore warrant careful interpretation of the data but would rather implicate rs11017848 as the primary driver than pleiotropic outlier for several reasons. First, we do not find evidence for pleiotropic effects of rs11017848 in the Rücker framework or when visually inspecting the funnel plots. Second, leave-one-out analysis has the disadvantage of forcing exclusion of genetic variants, resulting in a large percentage of reduction of the set of genetic variants when only three variants are included. Other outlier-proof methods, such as MR-Lasso, weighted median and MR contamination mixture model, showed results consistent with the inverse-variance weighted effect model. Third, we also found that genetically predicted fetuin-A increased type 2 diabetes risk in our Mendelian randomization analysis in which we used SNP selections based on the studies from Fisher et al*.*^8^ and Kröger et al*.*^10^, which did not include genetic variant rs11017848.

A biological explanation for the association between genetically predicted fetuin-A and type 2 diabetes might be found in the association between fetuin-A and free fatty acids. Previous experiments in adipocytes taken from mice and humans have shown that fetuin-A is required for free fatty acids to induce an inflammatory signaling pathway that results in insulin resistance^12^. A previous observational study from Harring et al.^13^ in 347 participants at high risk of cardiovascular disease found an interaction of fetuin-A and free fatty acids in determining insulin sensitivity, further supporting the theory that fetuin-A induces insulin insensitivity through free fatty acids.

In accordance with previous research^8,9^, the current Mendelian randomization analyses do not find evidence for an association with coronary artery disease or myocardial infarction in the unstratified population as a whole. MR sensitivity analysis indicated that the significant association between genetically predicted fetuin-A and myocardial infarction in the univariable IVW-MRE analysis is probably driven by one or several outliers. Regression to the mean due to a considerable larger sample size of the current study (Ncases = 20,864, Ncontrols = 391,580) compared to the positive EPIC Potsdam study (Ncases = 214, Ncontrols = 2197) might explain the discrepancy of results.

An additional explanation is that the effect of genetically predicted fetuin-A on cardiovascular disease development is conditional on patient characteristics and comorbidities. In the current study, we find evidence that genetically predicted fetuin-A increases coronary artery disease risk in individuals with type 2 diabetes, but we do not find an association between genetically predicted fetuin-A and coronary artery disease in participants without type 2 diabetes. This is in line with study from Laugsland et al.^9^, who described a lower CAD risk among individuals without type 2 diabetes and found a trend in the opposite direction among individuals with type 2 diabetes. We also find evidence that genetically predicted fetuin-A decreases myocardial infarction risk in women, but we do not find evidence for an association between genetically predicted fetuin-A and myocardial infarction in men. This might implicate that the development of acute coronary syndrome through the pathophysiologic pathway of ectopic calcification plays a more prominent role in women as compared to men.

In line with previous studies, we did not find evidence for an association between genetically predicted fetuin-A and any or ischemic stroke^8,9^. Stroke and ischemic stroke have a heterogenic pathophysiology, of which the hypothetical role of fetuin-A on calcification formation is only one of the potential causes. This might have limited the ability to analyze the role of genetically predicted fetuin-A on the development of calcification mediated stroke.

### Strengths and limitations

Strengths of the present study include the large sample size with a wide distribution in age, long and complete follow-up, extensive sensitivity analyses, and a large number of CVD events. Our study also has several limitations.

We did not have direct measurements of plasma fetuin-A and were therefore unable to validate instrument strength in the UK Biobank. We used common genetic variants that have consistently been found to be strongly associated with fetuin-A levels. One of these genetic variants, rs2248690, has a previously established biological mechanism through which it might alter fetuin-A^14^. The T-allele of rs2248690 was found to show a higher affinity to corepressor transcription factor AP-1 in human HepG2 cells. Increased affinity to transcription factor AP-1, a corepressor, would lead to lower levels of fetuin-A^14^. We note that the association of the other genetic variants with fetuin-A is statistical in nature, and in vitro studies are necessary to evaluate the biological mechanisms these genetic variants exert on fetuin-A. We therefore adopted a two-sample MR strategy, which decreases the chance of type 1 error rates due to potential weak-instrument bias, population stratification or correlated pleiotropy^15^.

The results of the interaction analysis should be interpreted with caution as only the SNP-outcome association was assessed due to the unavailability of fetuin-A in the UK Biobank. The fetuin-A associated genetic variants were not validated in the subgroups assessed in the interaction analyses, i.e. by sex or diabetes status, and we were therefore unable to assess potential weak-instrument bias in these subgroups. Future studies could perform an interaction Mendelian randomization using a Gene-by-Environment interactions framework^16^. The outcome cohort included individuals that were mainly, but not exclusively from European descent, and results are only generalizable to this group. We note that the more lenient clumping threshold that was applied for the sensitivity analyses could reintroduce confounding through inclusion of genetic variants in linkage disequilibrium. However, the results were generally consistent with the main analysis and could therefore be considered as further support.

Lastly, the current study is not interventional in design and the results should therefore be interpreted as unconfounded rather than causal estimates. The available data in this study does not allow for exploration of the gene-environment equivalence assumption. We stress that any claims of unconfounded association can only be made if interventions that alter fetuin-A equal the biological mechanisms in which fetuin-A associated genetic variants affect fetuin-A.

## Conclusion

In conclusion, we present evidence for an association between genetically predicted fetuin-A and type 2 diabetes. In addition, we find a conditional effect of genetically predicted fetuin-A on the development of coronary artery disease, where genetically predicted fetuin-A increases coronary artery disease risk in individuals with type 2 diabetes, whilst in those without type 2 diabetes there was no significant association between genetically predicted fetuin-A and coronary artery disease. Furthermore, higher genetically predicted fetuin-A reduces the risk of myocardial infarction in women, but we did not find evidence for an association between genetically predicted fetuin-A and myocardial infarction in men. Finally, we do not find evidence for an association between genetically predicted fetuin-A and stroke.

## Methods

### Study population

The UK Biobank study has been extensively described previously^17^. In brief, the UK Biobank study is a population-based prospective cohort study based in the United Kingdom. The study has recruited more than 500.000 individuals aged between 40 and 60 years from 2006 until 2010. All participants have given informed consent for the study^18^. The study has approval from the North West Multi-center Research Ethics Committee for the UK and was performed in accordance with the relevant guidelines and regulations^18^. A study protocol for the current article was not preregistered. This research has been conducted using the UK Biobank resource under application number 15031.

### Ascertainment of health outcomes

Self-reported diagnoses, medication use, and Hospital Episode Statistics data were used to define and capture prevalent and incident type 2 diabetes, coronary artery disease, myocardial infarction , stroke, and ischemic stroke diagnoses and events. Participant follow-up ended at their death or June 30th, 2020 for participants from England, October 31st, 2016 for participants from Scotland, or February 29th, 2016 for participants from Wales, whichever came first. Data on prevalent and incident disease was processed and extracted using the ukbpheno v1.0 package in R^19^.

### Genotyping and imputation

Genotyping of UK Biobank participants was performed using custom Affymetrix Axiom UK Biobank Lung Exome Variant Evaluation or UK Biobank arrays with > 95% shared content and imputed to merged UK10K and 1000 Genomes Phase 3 panels. The genotyping and imputation methods, as well as the arrays and quality control procedures have been described in detail previously^20^.

### Covariate definitions

Age was calculated based on the date of birth, which was acquired from central registry and updated by participant. Sex was obtained from the NHS at the central registry during the recruitment. The genotyping array defines whether the participants were genotyped by the Affymetrix UK Biobank Axiom array or Affymetrix UK BiLEVE Axiom array. The 30 principal components were based on a previous article from Bycroft et al. Additional cardiovascular risk factors are provided in Table 1, including body mass index, smoking status, hypertension, hyperlipidemia, and family history of cardiovascular disease. BMI was calculated based on measured length and weight. Smoking status is defined based on self-reported data and defined according to the American Heart Association 2020 Impact Goal guidelines on lifestyle. Five groups were determined derived from the questionnaire data, including “never or < 100 cigarettes”, “stopped > 12 months”, “stopped <  = 12 months”, “active smoking occasionally”, and “active smoking daily”. Participants were included in the “active smoking occasionally” group in case they answered to smoke occasionally or less than one cigarette per day, and in the “active smoking daily” when they answered daily. Systolic blood pressure values were obtained through two automated and/or two manual blood pressure measurements. The average value of all available blood pressure measurements was used per phenotype. Automated measurements were corrected according to previously described methodology^21^. Hyperlipidemia was defined on a combination of ICD10 and ICD9 codes, self-reported use of cholesterol lowering medication, and medication codes (Online Table 8). Family history of heart disease was based on self-reported data during the first visit, in which participants were asked if their father or mother suffered from any disease, including heart disease.

### Selection of fetuin-A SNPs

We considered SNPs associated with fetuin-A in previous MR studies^8–10^ or GWAS^11^ that had a minor allele frequency > 0.5%. SNP data of two genetic variants used in the MR study of Jensen et al.^22^ was obtained using a gene-centric 50K SNP array, which contained 13 variants at the *AHSG* locus. The association of these two genetic variants with fetuin-A were was assessed in up to 3093 individuals of European and African American descent^22^.SNP data of the four genetic variants used in the MR study of Fisher et al.^8^ was obtained in the HapMap 22/phaseII CEPH (Utah residents with ancestry from northern and western Europe) population data using Tagger software. The association of these four genetic variants with fetuin-A was assessed in up to 2197 individuals of European ancestry^8^. SNP data of the three genetic variants used in the MR study from Kröger et al*.* was obtained from was obtained analogous to the method from Fisher et al*.* The association of these three genetic variants with fetuin-A was assessed in up to 965 individuals of European ancestry. The GWAS from Jensen et al*.* on fetuin-A was performed in 9055 American individuals from European descent^11^.In case multiple studies highlighted the same genetic variant associated with fetuin-A, we brought forward the effect estimates with the lowest *P*-value.

We used the PLINK clumping procedure (v1.9) to prune genetic variants at a linkage disequilibrium of R^2^ < 0.01 within a 2.5 Mb window on either site, utilizing the UK Biobank reference panel^20,23^. This resulted in a total of four genetic variants which were taken forward for the main MR analyses (rs4917, rs5030023, rs11017848, rs1042464), of which rs1042464 was removed from the MR because the forward strand of this palindromic variant could not be correctly inferred. Considering the limited number of genetic variants included in the main Mendelian randomization analysis, we repeated our analysis using (a) a subset of 11 independent genetic variants at a more lenient clumping threshold of R^2^ = 0.05 (rs4917, rs9290835, rs4488820, rs13073740, rs4686799, rs843991, rs11918289, rs745588, rs11017848, rs4615068, and rs6809265), (b) a subset of genetic variants used in the study from Fisher et al.^8^ (rs4917, rs2070633, rs2248690, and rs2070635), and (c) a subset of variants used in the study of Kröger et al.^10^ (rs4917, rs2070633, rs2248690). An overview of the genetic variant selection method for the main and sensitivity analyses can be found in the flowchart provided in Supplementary Fig. 1.

We performed additional sensitivity analyses in order to compare current MR results with previous studies. We repeated the MR analyses using the effect sizes of the genetic variants on fetuin-A obtained from the Potsdam part of the EPIC-InterAct study^10^ instead of the effect sizes obtained from the meta-analysis of the CHARGE consortium^11^. The genetic variants used in the study from Kröger et al.^10^ were used (rs2070633, rs2248690, rs4917). Effect sizes of the genetic variants on type 2 diabetes were obtained in the UK Biobank, as described above.

Jensen et al.^11^ mentioned an additional conditional analysis resulting in 34 variants associated with fetuin-A independent of rs4917 in their paper. However, their report on this analysis was incomplete, as only 10 of these 34 variants were listed. These were 10 variants that were not previously found in the main GWAS. The conditioned effects of the original GWAS variants were not reported. This made it impossible to avoid potential overestimation of the effect and mixture of estimates from different models. To avoid these errors as well as a possible selection bias, we chose to exclude the conditional SNPs and kept only rs4917.

### Statistical analyses

#### Genetic variant-outcome association

To obtain the effect sizes of the genetic variant-outcome association, we performed logistic regression analyses fetuin-A associated SNPs and all health outcomes, including coronary artery disease, myocardial infarction, any stroke, ischemic stroke and type 2 diabetes. All logistic regression analyses were adjusted for age, sex, genotyping array, and the first 30 genetic principal components to adjust for population stratification. The genetic variant- exposure associations, obtained from the GWAS from Jensen et al.^11^, were also obtained from regression analyses corrected for age, sex, and principal components.

#### Mendelian randomization analyses

Our first objective was to assess the association of fetuin-A and with cardiovascular diseases and type 2 diabetes using a two-sample Mendelian randomization approach. Mendelian randomization analysis requires several assumptions to be fulfilled for correctly inferring a potential causal, or rather a generally unconfounded, estimate. In the case of the current study, these include (A) a strong and reliable association between the genetic variant and fetuin-A, (B) independence of unobserved confounding through fetuin-A, and C) the association between genetically predicted fetuin-A and the outcomes is caused through fetuin-A.

Weak-instrument bias was assessed by calculating the F-statistic using the following formula: *F* = *R*^2^(*n − *2)/(1* − R*^2^)*.* Here, *n* is the sample size used to obtain the fetuin-A estimates and R^2^ is the amount of variance in fetuin-A explained by the genetic variant^24^. An F-statistic < 10 was considered to indicate weak-instrument bias. MR-Steiger filtering was performed to assess reversed causation. Genetic variants with a significantly higher (*P* < 0.05) R^2^ for the outcome than for the fetuin-A were removed from further analyses^25^. The R^2^ for fetuin-A and the binary outcomes on the liability scale were calculated based on previously established formulae^26,27^.

MR estimates were obtained using an inverse variance weighted multiplicative random effects model (IVW-MRE). Potential pleiotropy was assessed using the Rucker framework^28^. Heterogeneity in the IVW estimates as indicated by a significant Cochran’s Q was (*P* < 0.05) in combination with a high *I*^2^ index (> 25%) was considered indicative of balanced horizontal pleiotropy^29^. We then fitted an MR-Egger model and calculated the heterogeneity in these estimates using Rucker’s Q. The MR-Egger model allows for a non-zero intercept and can therefore be used to assess unbalanced horizontal pleiotropy^30^. A significant Q-Q’ (*P* < 0.05), which is the difference between the Cochran’s Q and Rücker’s Q, in combination with a significant MR-Egger intercept (*P* < 0.05) was considered indicative of unbalanced horizontal pleiotropy. The MR-Egger test can provide a true causal estimate only under the under the general InSIDE (Instrument Strength Independent of Direct Effect) assumption and its flexibility in allowing for unbalanced horizontal pleiotropy generally comes at the cost of power to detect potential causal associations^30^. A high *I*^2^_GX_ (> 95%) was considered low risk of weak instrument bias within the MR-Egger estimates^31^. We report the IVW-MRE model as the main analysis in the scenario of balanced horizontal pleiotropy. The MR-Egger model was taken forward in the scenario of unbalanced horizontal pleiotropy.

Three additional sensitivity analyses were performed in the univariable MR setting. The MR-Lasso method has the ability to detect outliers and can provide consistent causal estimates in the scenario that a small portion of the genetic variants is invalid^32^. The weighed median approach is robust to more severe violations of the MR assumptions and provides a consistent estimate if up to half of the variants are invalid. Finally, the MR contamination mixture method was used to provide a consistent estimate if the plurality of the genetic variants is valid. In other words, it allows estimates a consistent estimate when this subset of valid genetic variants is not smaller than any subset of invalid genetic variants that assess the same genetic association^33^.

A more detailed explanation of MR assumptions and integration of the described methodology in a theoretical framework is provided is provided in Supplementary Figs. 6 and 7.

Genetic effect estimates are reported in odds ratios, since the SNP-outcome associations were obtained through logistic regression analyses. The MR analyses were considered significant at a Bonferroni corrected α = 0.05/5 outcomes. For the sensitivity MR analyses, we adapted a more lenient α = 0.05 to ascertain statistical significance considering these were performed to replicate the findings of the main analysis. Continuous variables are displayed as mean ± standard deviation when normally distributed and as median and interquartile ranges when skewed. Categorical variables are displayed as percentages. Regression analyses to obtain SNP-outcome associations were performed using statistical software STATA 15 (StataCorp LP). MR analyses were performed using R (version 4.0.5), the TwoSampleMR package 0.5.6^34^, MR-Lasso source code^32^, Mendelian Randomization^35^ (version 0.5.1).

#### Interaction analyses

Our second objective was to assess a comorbidity dependent effect exists of fetuin-A on health outcomes. We constructed weighted polygenetic risk scores of fetuin-A by summing the number of alleles (0, 1, or 2) for each individual after multiplication with the effect size between the genetic variant and fetuin-A. The genetic risk scores were constructed for the main analysis in which genetic variants were clumped at R^2^ < 0.01. In addition, the genetic risk scores were constructed using genetic variants selected according to the MR sensitivity analysis, including (a) a subset of 11 independent genetic variants at a more lenient clumping threshold of R^2^ = 0.05, (b) a subset of variants used in the study of Fisher et al*.*^8^, and (c) a subset of genetic variants used in the study from Kröger et al*.*^10^.

First, we assessed a type 2 diabetes effect of fetuin-A and cardiovascular outcomes, including coronary artery disease, myocardial infarction, any stroke, and ischemic stroke. The analyses included the interaction between the fetuin-A genetic risk score and type 2 diabetes in a logistic regression model. In this analysis we included only participants with type 2 diabetes diagnosis prior to the onset of the CVD to allow for a time interval in which a potential biological interaction with genetically predicted fetuin-A could occur.

Second, we assessed the interaction of fetuin-A with age, sex, BMI, hypertension and hyperlipidemia in the association with cardiovascular outcomes and type 2 diabetes. Participants were categorized based on age (higher or equal to 60 or below 60), BMI (> = 30 at baseline), hypertensive (systolic blood pressure >  = 140 mmHg or DBP >  = 90 mmHg) based on the average blood pressure of through two automated and/or two manual blood pressure measurements, as stated in the covariate section. Hyperlipidemic if individuals had a history of hyperlipidemia based on ICD10 and ICD9 codes (Online Table 6). Hyperlipidemia was defined on a combination of ICD10 and ICD9 codes, self-reported use of cholesterol lowering medication, and medication codes (Online Table 8).

Thirdly, we assessed potential three-way interactions between fetuin-A, sex, and type 2 diabetes in the association with cardiovascular outcomes. The analyses included the three-way interaction between the fetuin-A genetic risk score, sex, and type 2 diabetes in a logistic regression model.

Regression analyses to obtain SNP-outcome associations were performed using statistical software STATA 15 (StataCorp LP). For the interaction analysis we adapted α = 0.05 to ascertain statistical significance.

### Supplementary Information

Supplementary Information 1.

Supplementary Information 2.

## Data Availability

All data supporting the findings described in this manuscript are available in the article and its Supplementary Information files.

## Abbreviations

CAD: Coronary artery disease
CVD: Cardiovascular disease
MI: Myocardial infarction
MR: Mendelian randomization
