# Supplementary material for: Fetuin-A and its genetic association with cardiometabolic disease
Source: Sci Rep. 2023 Dec 6;13:21469. doi: 10.1038/s41598-023-48600-9 (PMC10697970; doi:10.1038/s41598-023-48600-9)
Supplement: Supplementary file 1 — Supplementary Information 1. [file 41598_2023_48600_MOESM1_ESM.docx]

**Fetuin-A and its association with coronary artery disease, myocardial infarction, and ischemic stroke**

Lawien Al Ali, Yordi J. van de Vegte, M. Abdullah Said, Tom Hendriks, Ming Wai Yeung, Erik Lipsic, Pim van der Harst

**Supplementary information**

**Supplementary Figure 1: Flowchart of the genetic variant selection for the main and sensitivity analyses
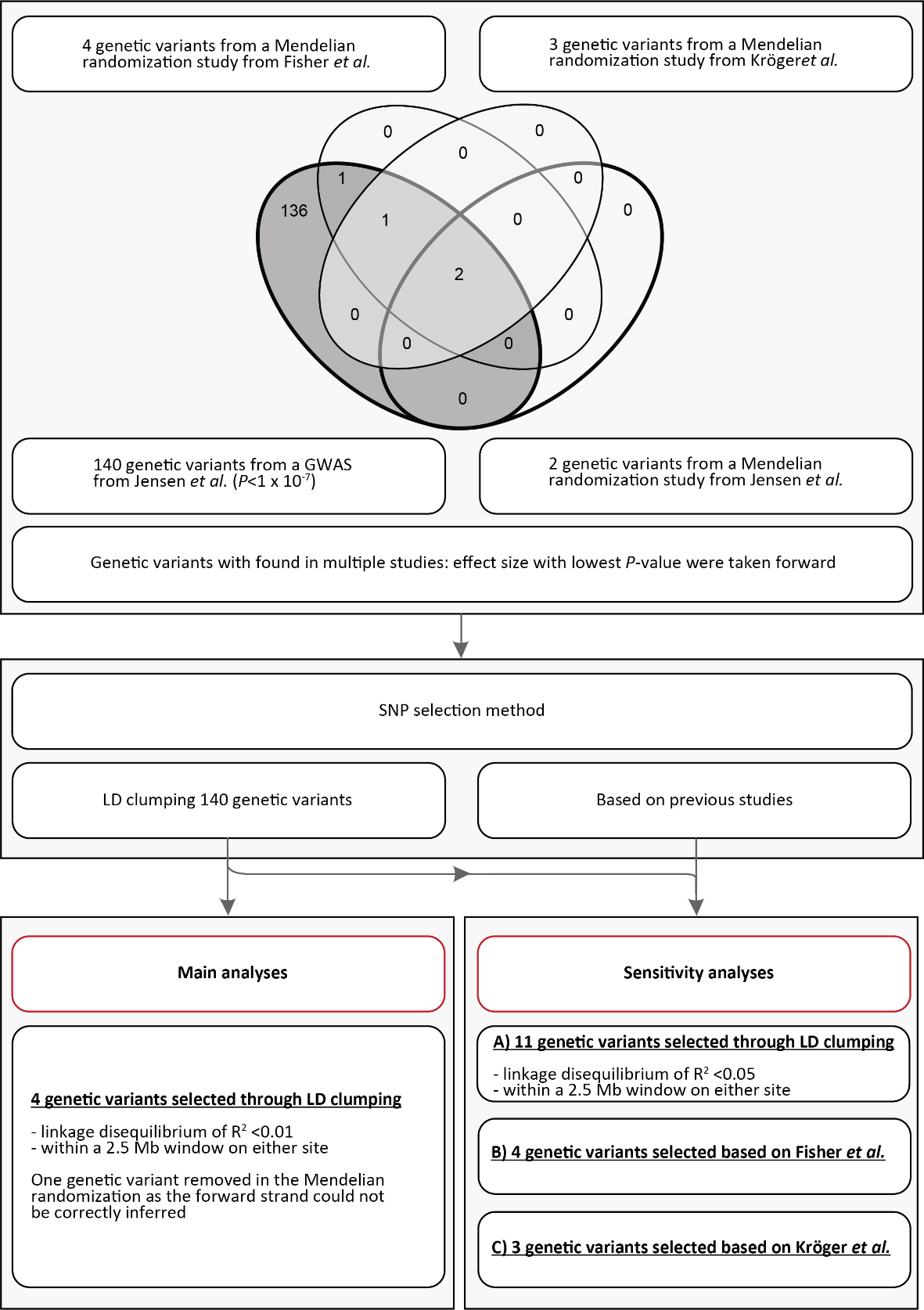
**

*Flowchart of the genetic variant selection for the main and sensitivity analyses. Shown in the upper panel is a Venn diagram with the genetic variant overlap between previous genome-wide association and Mendelian randomization studies. In case multiple studies highlighted the same genetic variant associated with fetuin-A, we brought forward the effect estimates with the lowest P-value. The middle panel shows the two methods for genetic variant selection, i.e., clumping all variants or genetic variant selection based on previous studies. The lower panels show the resulting number of genetic variants per strategy. The left panel shows the number of genetic variants selected for the main analyses, the right panel the number of genetic variants selected for the multiple sensitivity analyses. The sensitivity analysis includes* *a) a subset of 11 independent genetic variants at a more lenient clumping threshold of R2 = 0.05, b) a subset of variants used in the study of Fisher et al., and c) a subset of genetic variants used in the study from Kröger et al. SNP information*

**Supplementary Figure 2:** Scatterplots, forest plots, funnel plots, and leave-one-out plots of the Mendelian randomization analyses of genetically predicted fetuin-A with cardiovascular diseases and type 2 diabetes, using all independent genetic variants at R^2^=0.01.

A) Fetuin-A – coronary artery disease

*
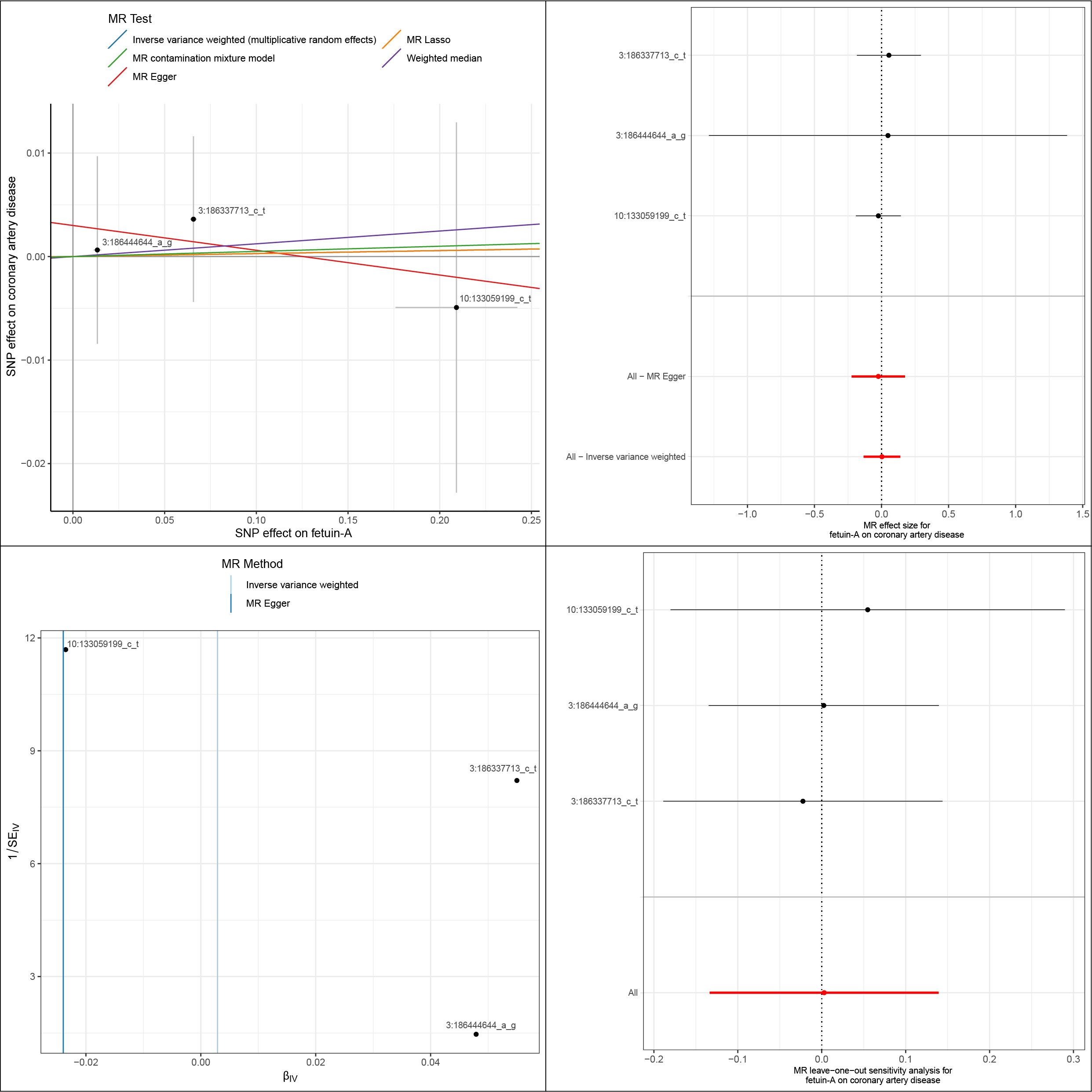
*

*Scatterplots, forest plots, funnel plots, and leave-one-out plots of the Mendelian randomization analyses between genetically predicted fetuin-A and coronary artery disease within the UK Biobank using independent genetic variants at R^2^=0.01. A) Scatter plot: the variants’ effect size and standard error on fetuin-A are displayed on the X-axis, the variants’ effect size and standard error on the outcomes are displayed on the Y-axis. The blue line is the regression line of the inverse variance weighted multiplicative random effects meta-analysis, the green line of the MR contamination mixture model, the red line of the MR-Egger analysis, the orange line of the MR Lasso method and the purple line of the weighted median method. Top right: forest plot. The effect size in beta and SE are displayed on the X-axis, the genetic variants or statistic test are displayed on the X-axis. Wald estimates are displayed in black, pooled effect estimates in red. Bottom left: funnel plot. The beta of the Wald estimate per SNP is displayed on the X-axis, the Y-axis displays 1/standard error of the Wald estimate. The light blue line demonstrates the results of the inverse variance weighted analysis, dark blue the results of the MR-Egger analysis. Bottom right: leave-one-out plot. The effect size in beta and SE are displayed on the X-axis, the genetic variants left out or the pooled inverse variance weighted effect estimate of all included SNPs are displayed on the Y-axis. SNP denotes single nucleotide polymorphism, MR denotes Mendelian randomization.*

B) Fetuin-A – myocardial infarction

**
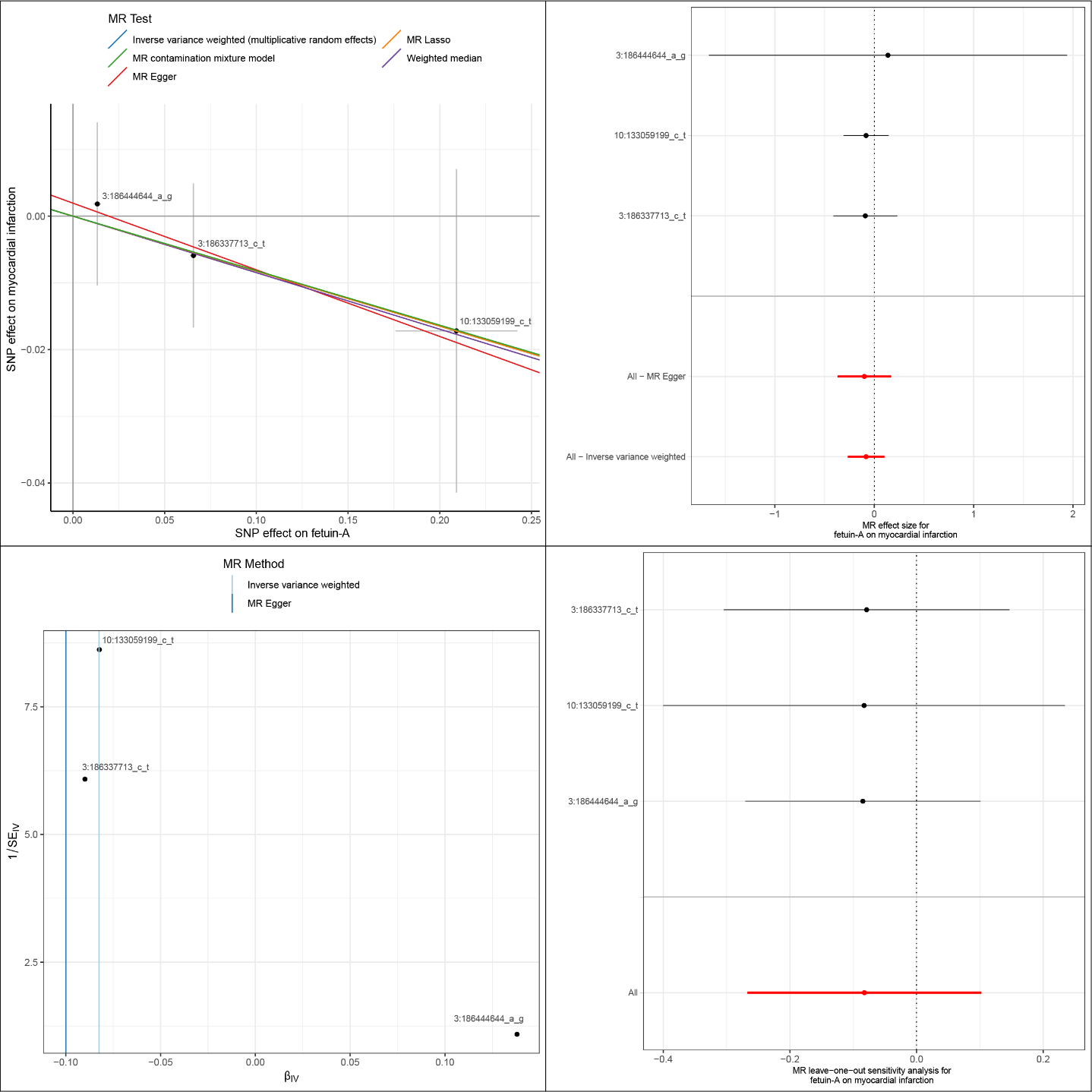
**

*Scatterplots, forest plots, funnel plots, and leave-one-out plots of the Mendelian randomization analyses between genetically predicted fetuin-A and myocardial infarction within the UK Biobank using independent genetic variants at R^2^=0.01. A) Scatter plot: the variants’ effect size and standard error on fetuin-A are displayed on the X-axis, the variants’ effect size and standard error on the outcomes are displayed on the Y-axis. The blue line is the regression line of the inverse variance weighted multiplicative random effects meta-analysis, the green line of the MR contamination mixture model, the red line of the MR-Egger analysis, the orange line of the MR Lasso method and the purple line of the weighted median method. Top right: forest plot. The effect size in beta and SE are displayed on the X-axis, the genetic variants or statistic test are displayed on the X-axis. Wald estimates are displayed in black, pooled effect estimates in red. Bottom left: funnel plot. The beta of the Wald estimate per SNP is displayed on the X-axis, the Y-axis displays 1/standard error of the Wald estimate. The light blue line demonstrates the results of the inverse variance weighted analysis, dark blue the results of the MR-Egger analysis. Bottom right: leave-one-out plot. The effect size in beta and SE are displayed on the X-axis, the genetic variants left out or the pooled inverse variance weighted effect estimate of all included SNPs are displayed on the Y-axis. SNP denotes single nucleotide polymorphism, MR denotes Mendelian randomization.*

C) Fetuin-A – any stroke

**
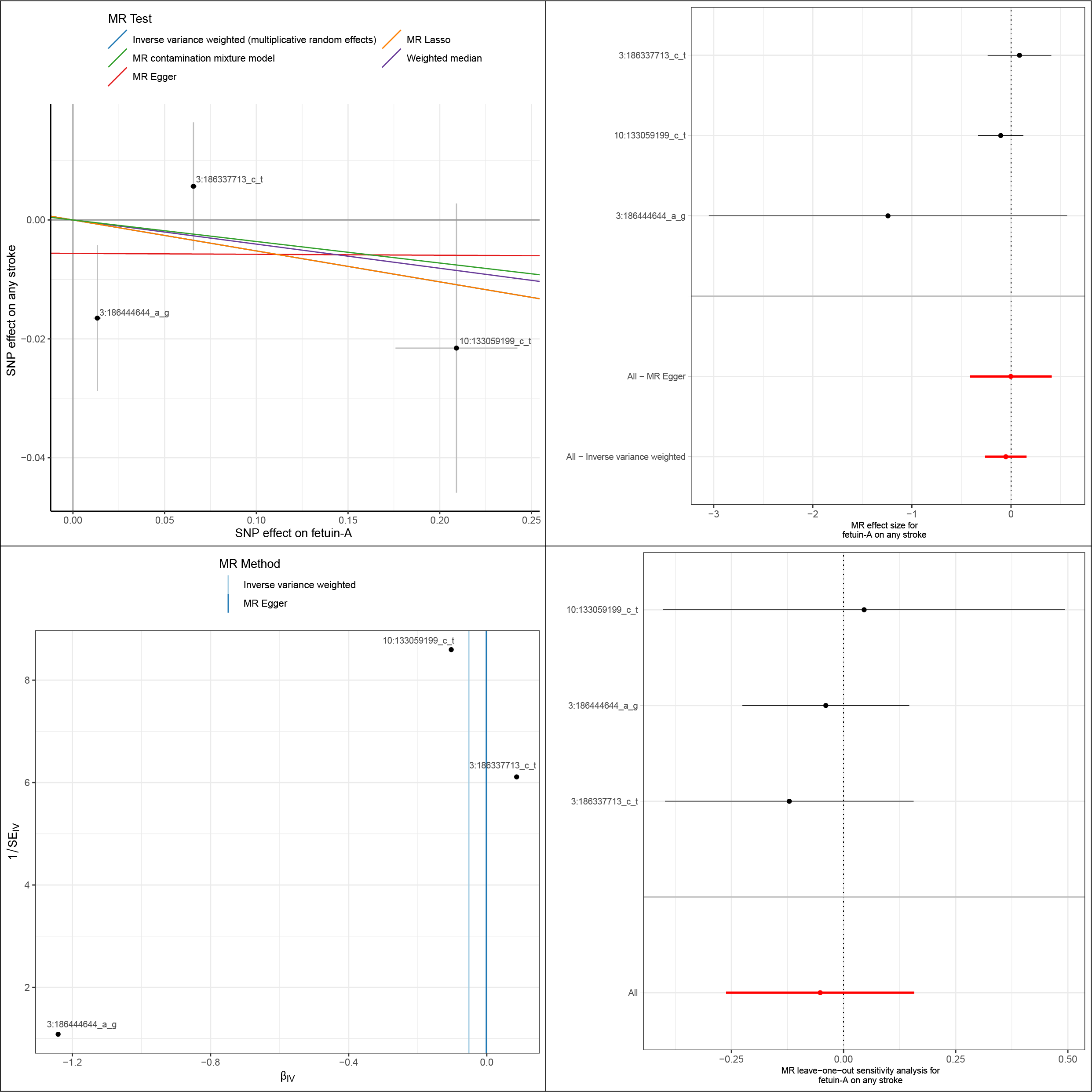
**

*Scatterplots, forest plots, funnel plots, and leave-one-out plots of the Mendelian randomization analyses between genetically predicted fetuin-A and any stroke within the UK Biobank using independent genetic variants at R^2^=0.01. A) Scatter plot: the variants’ effect size and standard error on fetuin-A are displayed on the X-axis, the variants’ effect size and standard error on the outcomes are displayed on the Y-axis. The blue line is the regression line of the inverse variance weighted multiplicative random effects meta-analysis, the green line of the MR contamination mixture model, the red line of the MR-Egger analysis, the orange line of the MR Lasso method and the purple line of the weighted median method. Top right: forest plot. The effect size in beta and SE are displayed on the X-axis, the genetic variants or statistic test are displayed on the X-axis. Wald estimates are displayed in black, pooled effect estimates in red. Bottom left: funnel plot. The beta of the Wald estimate per SNP is displayed on the X-axis, the Y-axis displays 1/standard error of the Wald estimate. The light blue line demonstrates the results of the inverse variance weighted analysis, dark blue the results of the MR-Egger analysis. Bottom right: leave-one-out plot. The effect size in beta and SE are displayed on the X-axis, the genetic variants left out or the pooled inverse variance weighted effect estimate of all included SNPs are displayed on the Y-axis. SNP denotes single nucleotide polymorphism, MR denotes Mendelian randomization.*

D) Fetuin-A – any ischemic stroke

**
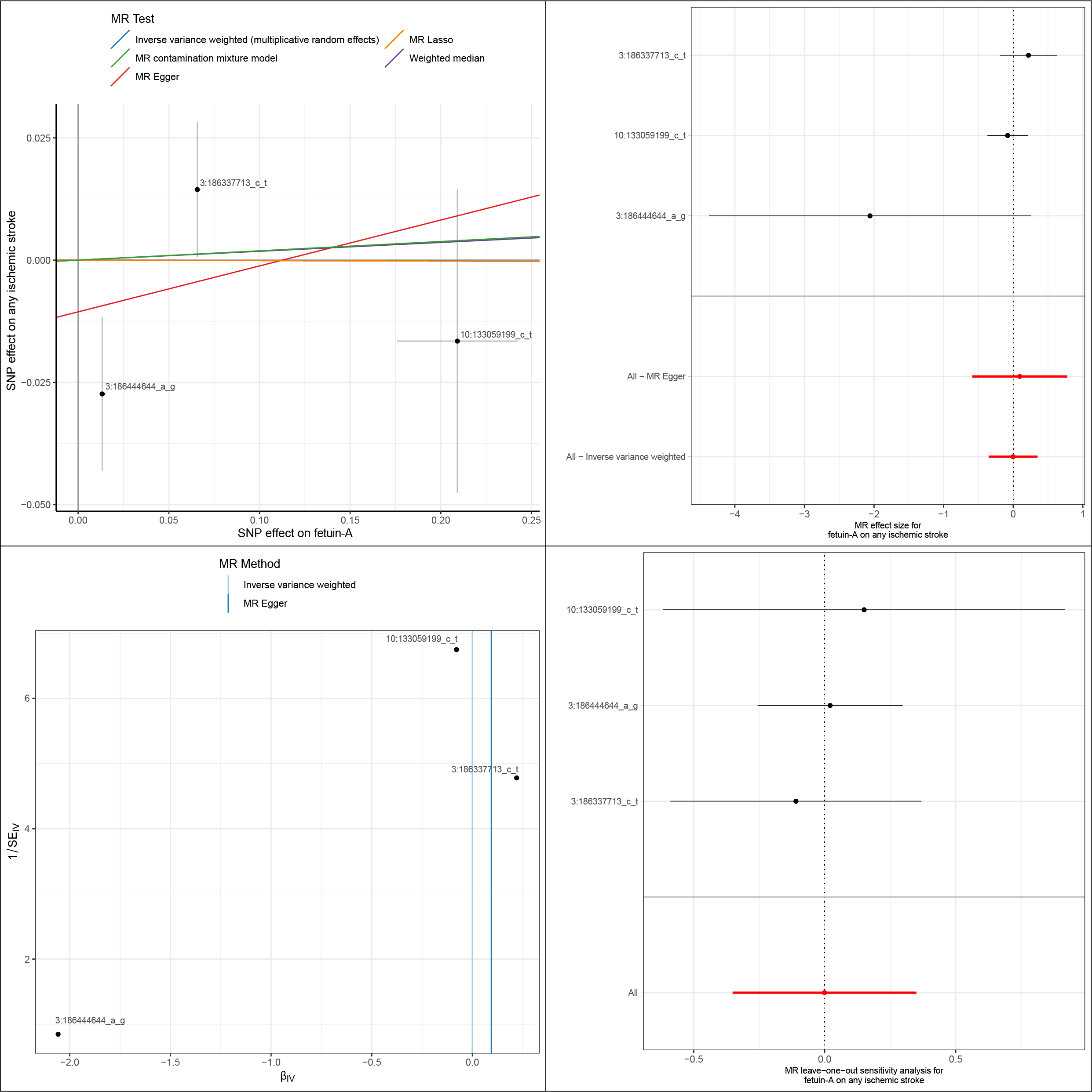
**

*Scatterplots, forest plots, funnel plots, and leave-one-out plots of the Mendelian randomization analyses between genetically predicted fetuin-A and any ischemic stroke within the UK Biobank using independent genetic variants at R^2^=0.01. A) Scatter plot: the variants’ effect size and standard error on fetuin-A are displayed on the X-axis, the variants’ effect size and standard error on the outcomes are displayed on the Y-axis. The blue line is the regression line of the inverse variance weighted multiplicative random effects meta-analysis, the green line of the MR contamination mixture model, the red line of the MR-Egger analysis, the orange line of the MR Lasso method and the purple line of the weighted median method. Top right: forest plot. The effect size in beta and SE are displayed on the X-axis, the genetic variants or statistic test are displayed on the X-axis. Wald estimates are displayed in black, pooled effect estimates in red. Bottom left: funnel plot. The beta of the Wald estimate per SNP is displayed on the X-axis, the Y-axis displays 1/standard error of the Wald estimate. The light blue line demonstrates the results of the inverse variance weighted analysis, dark blue the results of the MR-Egger analysis. Bottom right: leave-one-out plot. The effect size in beta and SE are displayed on the X-axis, the genetic variants left out or the pooled inverse variance weighted effect estimate of all included SNPs are displayed on the Y-axis. SNP denotes single nucleotide polymorphism, MR denotes Mendelian randomization.*

E) Fetuin-A – type 2 diabetes

**
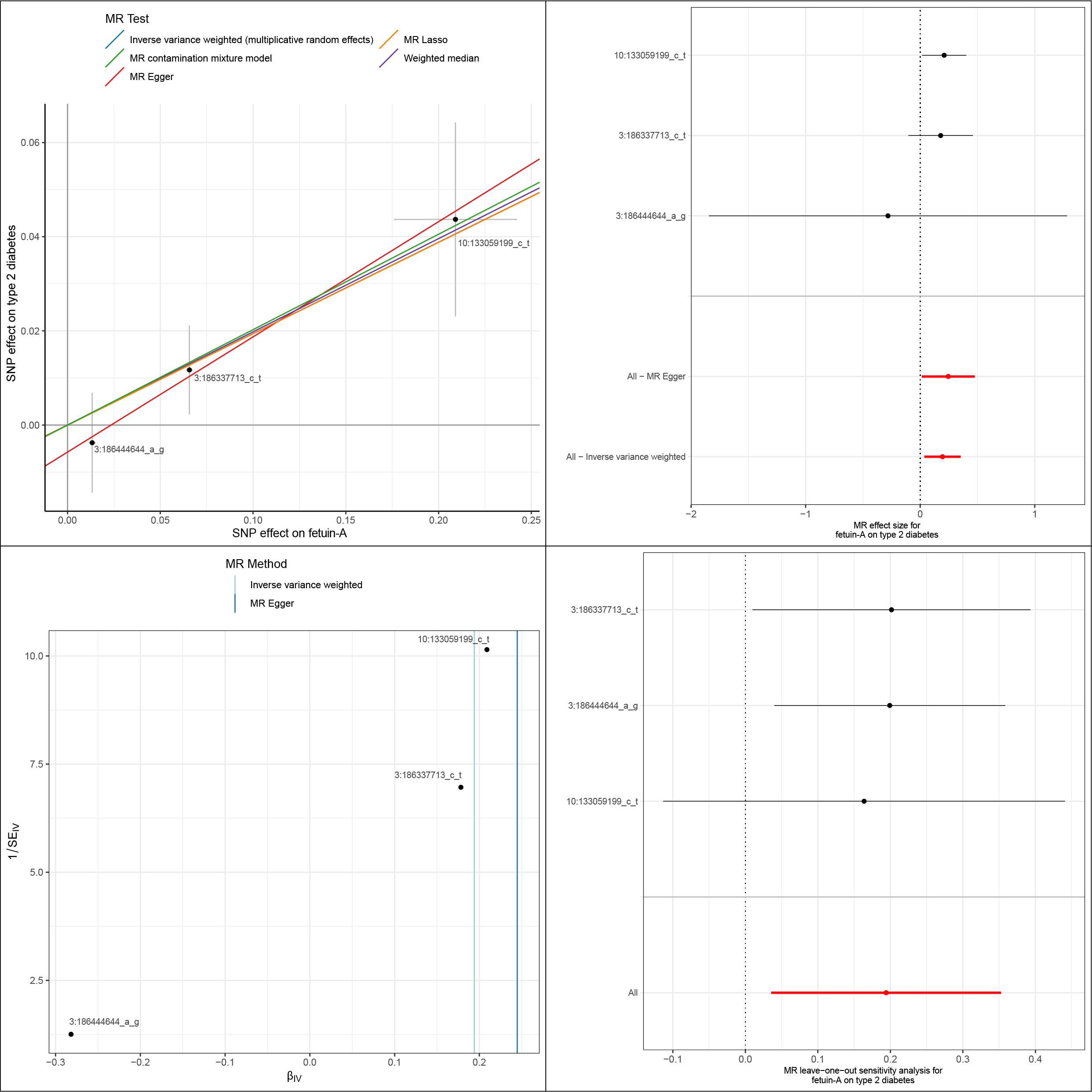
**

*Scatterplots, forest plots, funnel plots, and leave-one-out plots of the Mendelian randomization analyses between genetically predicted fetuin-A and type 2 diabetes within the UK Biobank using independent genetic variants at R^2^=0.01. A) Scatter plot: the variants’ effect size and standard error on fetuin-A are displayed on the X-axis, the variants’ effect size and standard error on the outcomes are displayed on the Y-axis. The blue line is the regression line of the inverse variance weighted multiplicative random effects meta-analysis, the green line of the MR contamination mixture model, the red line of the MR-Egger analysis, the orange line of the MR Lasso method and the purple line of the weighted median method. Top right: forest plot. The effect size in beta and SE are displayed on the X-axis, the genetic variants or statistic test are displayed on the X-axis. Wald estimates are displayed in black, pooled effect estimates in red. Bottom left: funnel plot. The beta of the Wald estimate per SNP is displayed on the X-axis, the Y-axis displays 1/standard error of the Wald estimate. The light blue line demonstrates the results of the inverse variance weighted analysis, dark blue the results of the MR-Egger analysis. Bottom right: leave-one-out plot. The effect size in beta and SE are displayed on the X-axis, the genetic variants left out or the pooled inverse variance weighted effect estimate of all included SNPs are displayed on the Y-axis. SNP denotes single nucleotide polymorphism, MR denotes Mendelian randomization.*

**Supplementary Figure 3:** Scatterplots, forest plots, funnel plots, and leave-one-out plots of the Mendelian randomization analyses of genetically predicted fetuin-A with cardiovascular diseases and type 2 diabetes, using all independent genetic variants at R^2^=0.05.

A) Fetuin-A – coronary artery disease

**
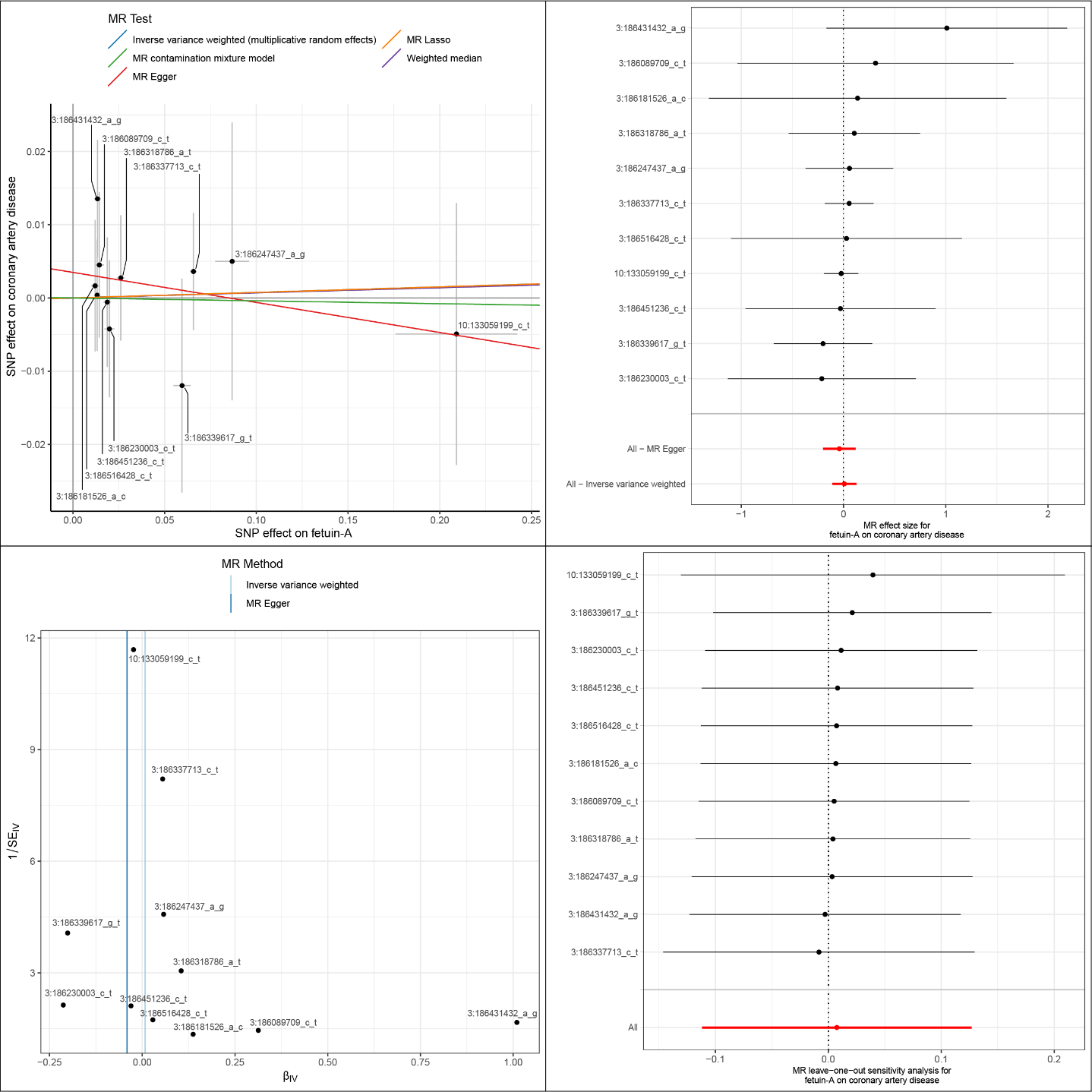
**

*Scatterplots, forest plots, funnel plots, and leave-one-out plots of the Mendelian randomization analyses between genetically predicted fetuin-A and coronary artery disease within the UK Biobank using independent genetic variants at R^2^=0.05. A) Scatter plot: the variants’ effect size and standard error on fetuin-A are displayed on the X-axis, the variants’ effect size and standard error on the outcomes are displayed on the Y-axis. The blue line is the regression line of the inverse variance weighted multiplicative random effects meta-analysis, the green line of the MR contamination mixture model, the red line of the MR-Egger analysis, the orange line of the MR Lasso method and the purple line of the weighted median method. Top right: forest plot. The effect size in beta and SE are displayed on the X-axis, the genetic variants or statistic test are displayed on the X-axis. Wald estimates are displayed in black, pooled effect estimates in red. Bottom left: funnel plot. The beta of the Wald estimate per SNP is displayed on the X-axis, the Y-axis displays 1/standard error of the Wald estimate. The light blue line demonstrates the results of the inverse variance weighted analysis, dark blue the results of the MR-Egger analysis. Bottom right: leave-one-out plot. The effect size in beta and SE are displayed on the X-axis, the genetic variants left out or the pooled inverse variance weighted effect estimate of all included SNPs are displayed on the Y-axis. SNP denotes single nucleotide polymorphism, MR denotes Mendelian randomization.*

B) Fetuin-A – myocardial infarction

*
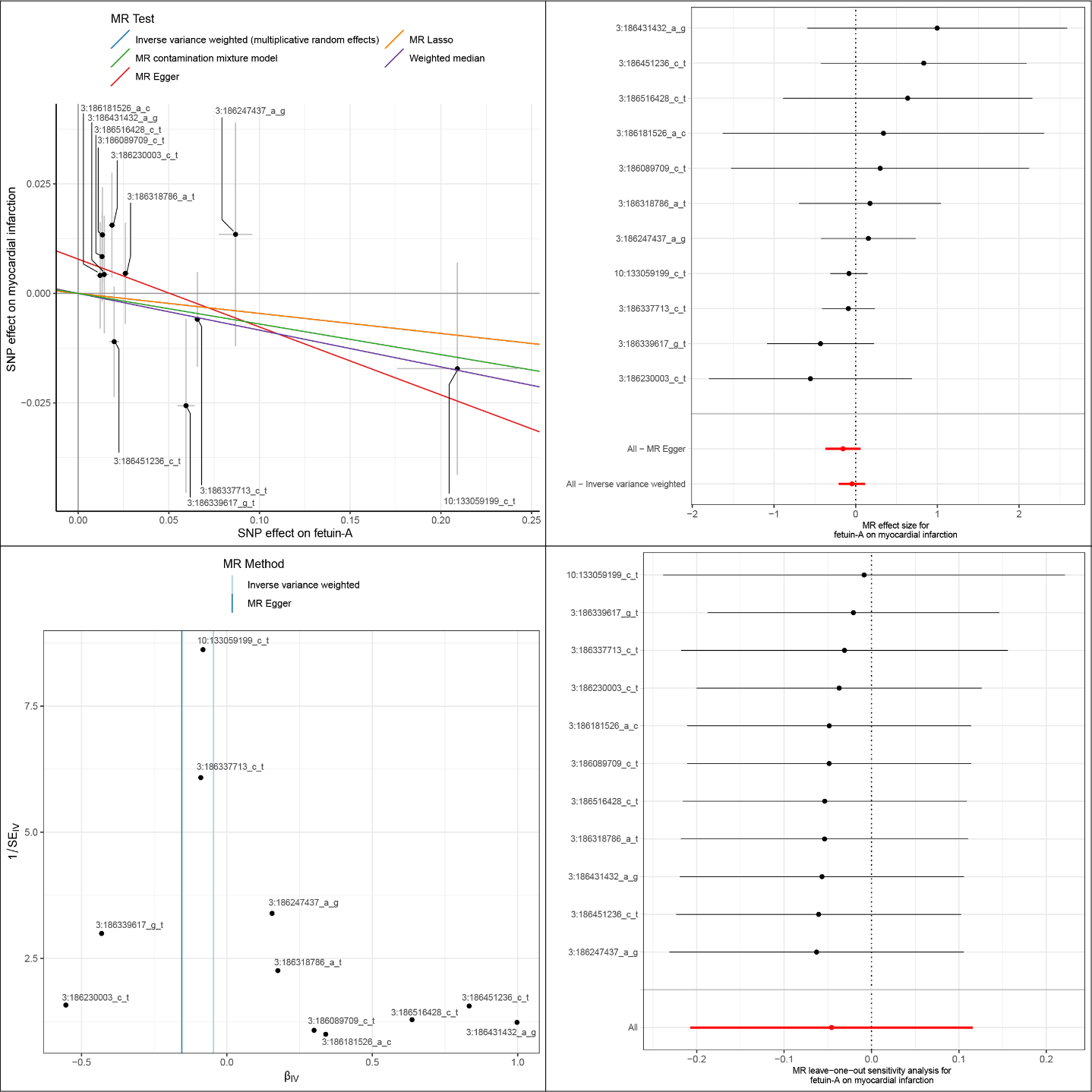
*

*Scatterplots, forest plots, funnel plots, and leave-one-out plots of the Mendelian randomization analyses between genetically predicted fetuin-A and myocardial infarction within the UK Biobank using independent genetic variants at R^2^=0.05. A) Scatter plot: the variants’ effect size and standard error on fetuin-A are displayed on the X-axis, the variants’ effect size and standard error on the outcomes are displayed on the Y-axis. The blue line is the regression line of the inverse variance weighted multiplicative random effects meta-analysis, the green line of the MR contamination mixture model, the red line of the MR-Egger analysis, the orange line of the MR Lasso method and the purple line of the weighted median method. Top right: forest plot. The effect size in beta and SE are displayed on the X-axis, the genetic variants or statistic test are displayed on the X-axis. Wald estimates are displayed in black, pooled effect estimates in red. Bottom left: funnel plot. The beta of the Wald estimate per SNP is displayed on the X-axis, the Y-axis displays 1/standard error of the Wald estimate. The light blue line demonstrates the results of the inverse variance weighted analysis, dark blue the results of the MR-Egger analysis. Bottom right: leave-one-out plot. The effect size in beta and SE are displayed on the X-axis, the genetic variants left out or the pooled inverse variance weighted effect estimate of all included SNPs are displayed on the Y-axis. SNP denotes single nucleotide polymorphism, MR denotes Mendelian randomization.*

C) Fetuin-A – any stroke

**
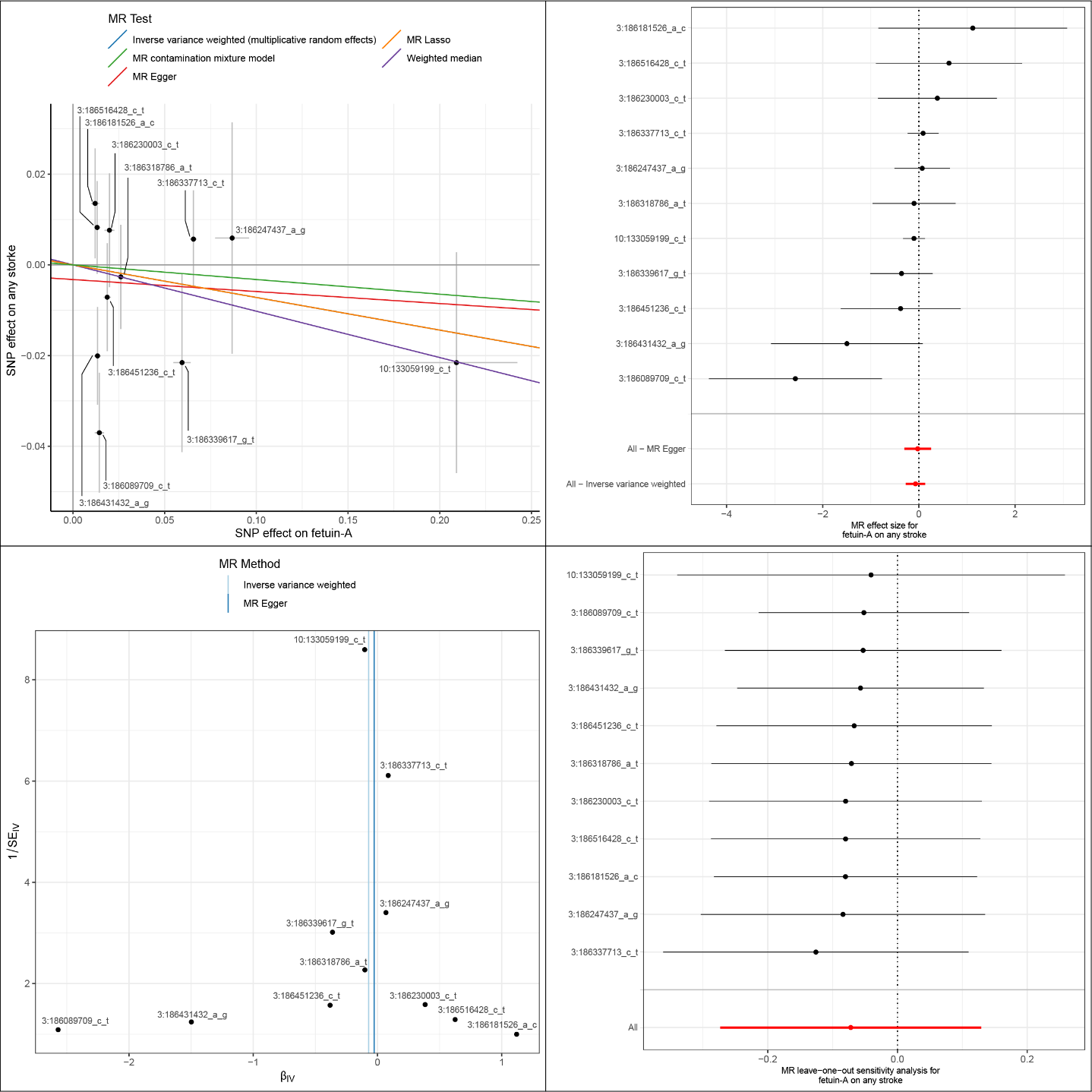
**

*Scatterplots, forest plots, funnel plots, and leave-one-out plots of the Mendelian randomization analyses between genetically predicted fetuin-A and any stroke within the UK Biobank using independent genetic variants at R^2^=0.01. A) Scatter plot: the variants’ effect size and standard error on fetuin-A are displayed on the X-axis, the variants’ effect size and standard error on the outcomes are displayed on the Y-axis. The blue line is the regression line of the inverse variance weighted multiplicative random effects meta-analysis, the green line of the MR contamination mixture model, the red line of the MR-Egger analysis, the orange line of the MR Lasso method and the purple line of the weighted median method. Top right: forest plot. The effect size in beta and SE are displayed on the X-axis, the genetic variants or statistic test are displayed on the X-axis. Wald estimates are displayed in black, pooled effect estimates in red. Bottom left: funnel plot. The beta of the Wald estimate per SNP is displayed on the X-axis, the Y-axis displays 1/standard error of the Wald estimate. The light blue line demonstrates the results of the inverse variance weighted analysis, dark blue the results of the MR-Egger analysis. Bottom right: leave-one-out plot. The effect size in beta and SE are displayed on the X-axis, the genetic variants left out or the pooled inverse variance weighted effect estimate of all included SNPs are displayed on the Y-axis. SNP denotes single nucleotide polymorphism, MR denotes Mendelian randomization.*

D) Fetuin-A – any ischemic stroke

**
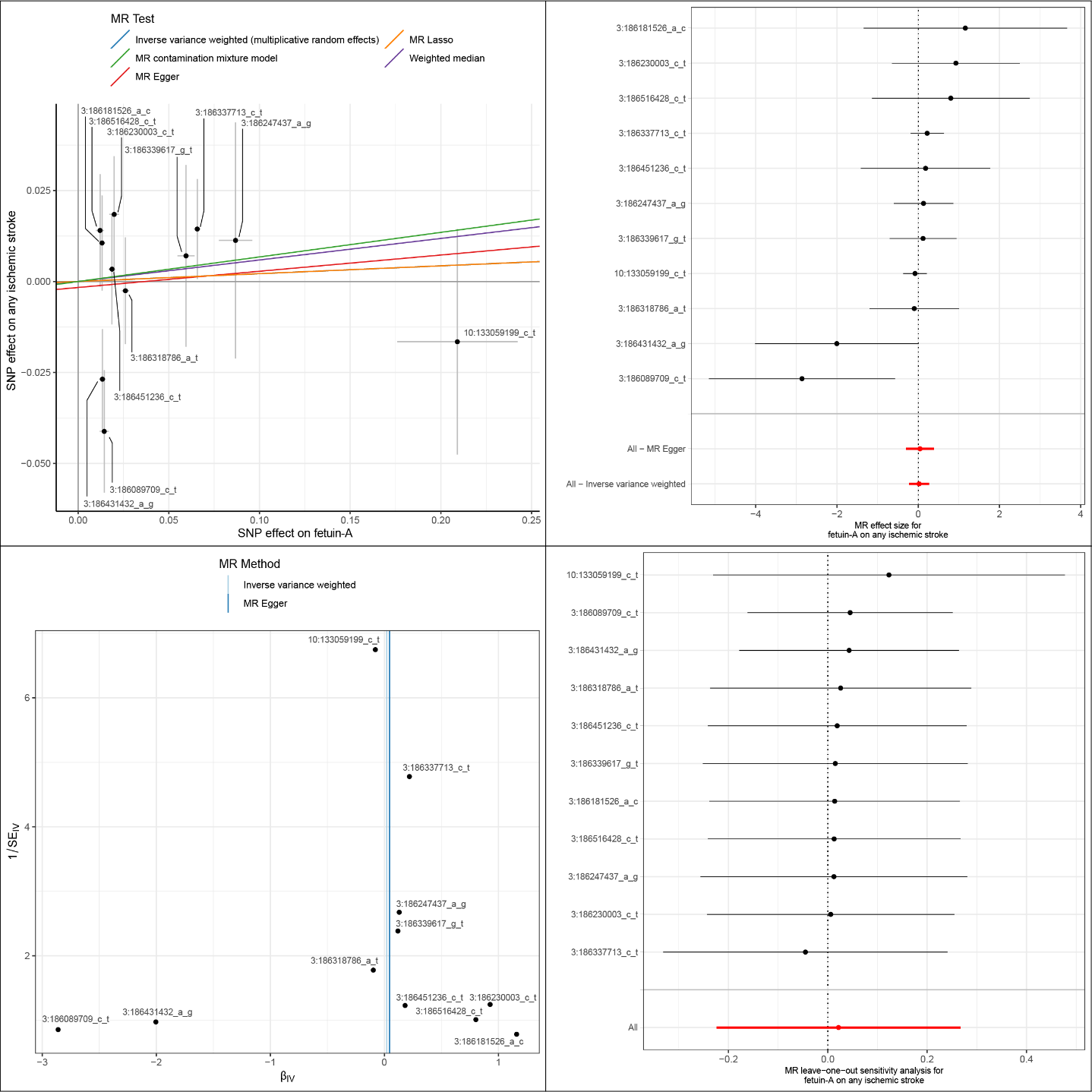
**

*Scatterplots, forest plots, funnel plots, and leave-one-out plots of the Mendelian randomization analyses between genetically predicted fetuin-A and any ischemic stroke within the UK Biobank using independent genetic variants at R^2^=0.05. A) Scatter plot: the variants’ effect size and standard error on fetuin-A are displayed on the X-axis, the variants’ effect size and standard error on the outcomes are displayed on the Y-axis. The blue line is the regression line of the inverse variance weighted multiplicative random effects meta-analysis, the green line of the MR contamination mixture model, the red line of the MR-Egger analysis, the orange line of the MR Lasso method and the purple line of the weighted median method. Top right: forest plot. The effect size in beta and SE are displayed on the X-axis, the genetic variants or statistic test are displayed on the X-axis. Wald estimates are displayed in black, pooled effect estimates in red. Bottom left: funnel plot. The beta of the Wald estimate per SNP is displayed on the X-axis, the Y-axis displays 1/standard error of the Wald estimate. The light blue line demonstrates the results of the inverse variance weighted analysis, dark blue the results of the MR-Egger analysis. Bottom right: leave-one-out plot. The effect size in beta and SE are displayed on the X-axis, the genetic variants left out or the pooled inverse variance weighted effect estimate of all included SNPs are displayed on the Y-axis. SNP denotes single nucleotide polymorphism, MR denotes Mendelian randomization.*

E) Fetuin-A – type 2 diabetes

**
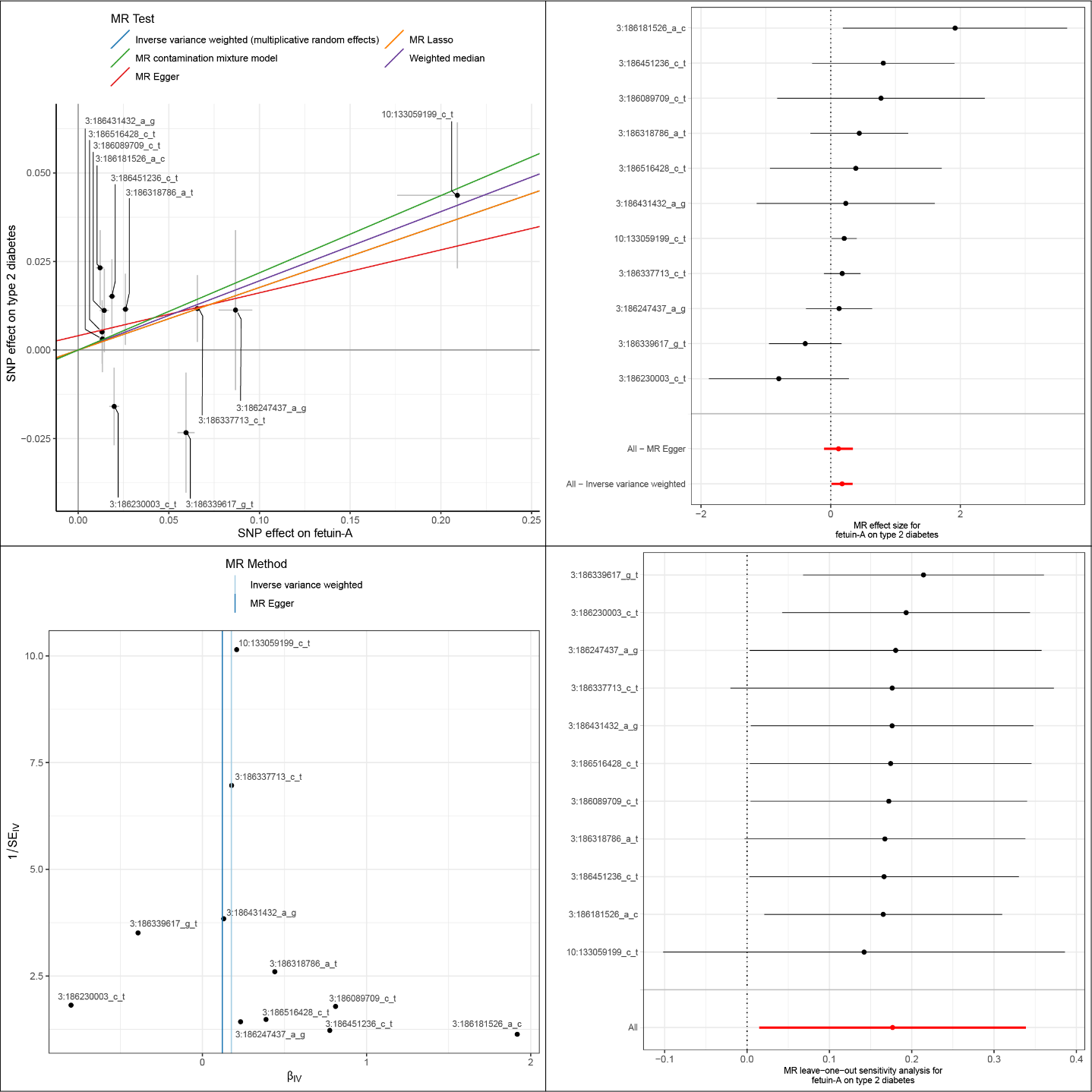
**

*Scatterplots, forest plots, funnel plots, and leave-one-out plots of the Mendelian randomization analyses between genetically predicted fetuin-A and type 2 diabetes within the UK Biobank using independent genetic variants at R^2^=0.05. A) Scatter plot: the variants’ effect size and standard error on fetuin-A are displayed on the X-axis, the variants’ effect size and standard error on the outcomes are displayed on the Y-axis. The blue line is the regression line of the inverse variance weighted multiplicative random effects meta-analysis, the green line of the MR contamination mixture model, the red line of the MR-Egger analysis, the orange line of the MR Lasso method and the purple line of the weighted median method. Top right: forest plot. The effect size in beta and SE are displayed on the X-axis, the genetic variants or statistic test are displayed on the X-axis. Wald estimates are displayed in black, pooled effect estimates in red. Bottom left: funnel plot. The beta of the Wald estimate per SNP is displayed on the X-axis, the Y-axis displays 1/standard error of the Wald estimate. The light blue line demonstrates the results of the inverse variance weighted analysis, dark blue the results of the MR-Egger analysis. Bottom right: leave-one-out plot. The effect size in beta and SE are displayed on the X-axis, the genetic variants left out or the pooled inverse variance weighted effect estimate of all included SNPs are displayed on the Y-axis. SNP denotes single nucleotide polymorphism, MR denotes Mendelian randomization.*

**Supplementary Figure 4:** Scatterplots, forest plots, funnel plots, and leave-one-out plots of the Mendelian randomization analyses of genetically predicted fetuin-A with cardiovascular diseases and type 2 diabetes, using genetic variants used in the study of Fisher *et al*.

A) Fetuin-A – coronary artery disease


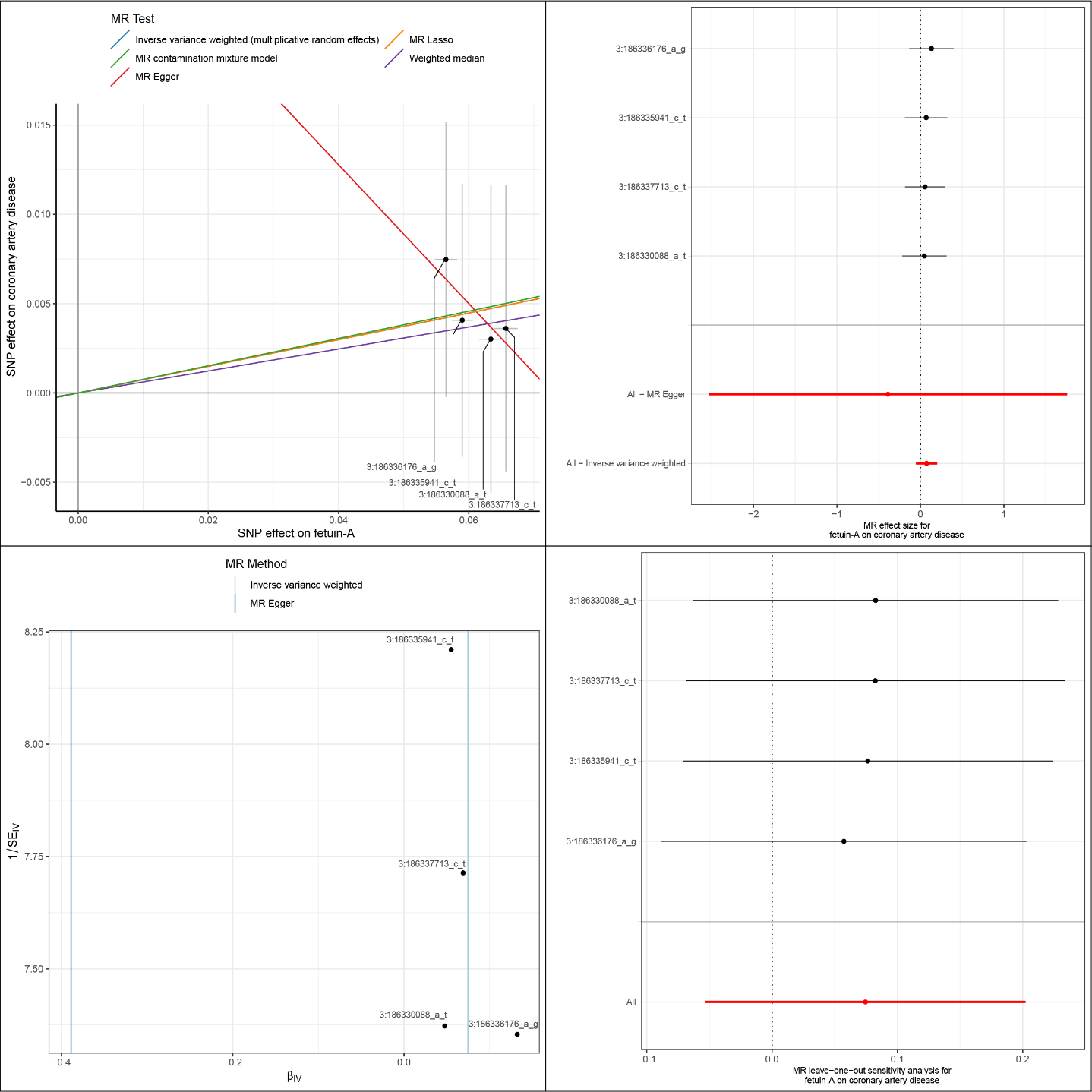


*Scatterplots, forest plots, funnel plots, and leave-one-out plots of the Mendelian randomization analyses between genetically predicted fetuin-A and coronary artery disease within the UK Biobank using the genetic variants used in the study from Fisher et al. A) Scatter plot: the variants’ effect size and standard error on fetuin-A are displayed on the X-axis, the variants’ effect size and standard error on the outcomes are displayed on the Y-axis. The blue line is the regression line of the inverse variance weighted multiplicative random effects meta-analysis, the green line of the MR contamination mixture model, the red line of the MR-Egger analysis, the orange line of the MR Lasso method and the purple line of the weighted median method. Top right: forest plot. The effect size in beta and SE are displayed on the X-axis, the genetic variants or statistic test are displayed on the X-axis. Wald estimates are displayed in black, pooled effect estimates in red. Bottom left: funnel plot. The beta of the Wald estimate per SNP is displayed on the X-axis, the Y-axis displays 1/standard error of the Wald estimate. The light blue line demonstrates the results of the inverse variance weighted analysis, dark blue the results of the MR-Egger analysis. Bottom right: leave-one-out plot. The effect size in beta and SE are displayed on the X-axis, the genetic variants left out or the pooled inverse variance weighted effect estimate of all included SNPs are displayed on the Y-axis. SNP denotes single nucleotide polymorphism, MR denotes Mendelian randomization.*

B) Fetuin-A – myocardial infarction

**
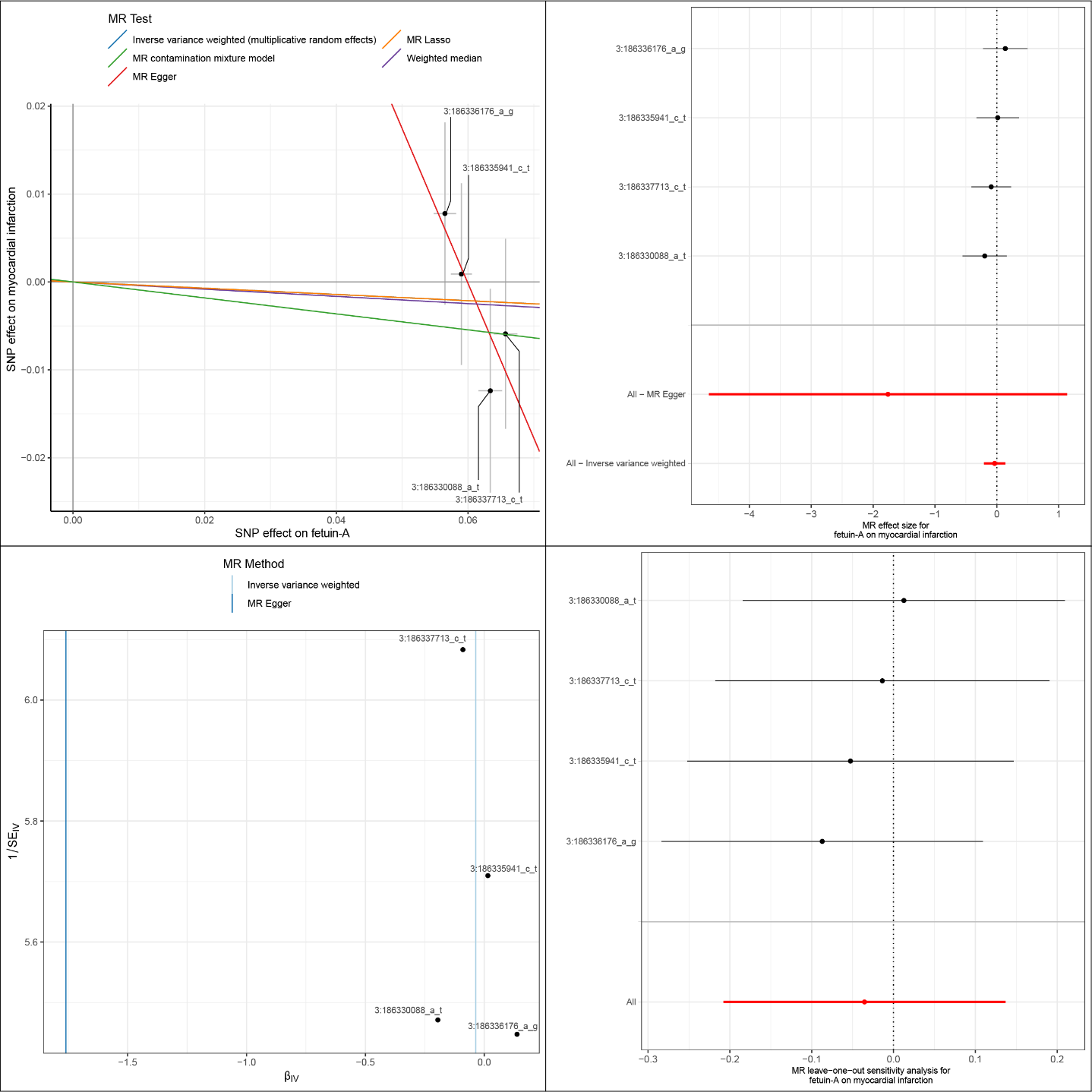
**

*Scatterplots, forest plots, funnel plots, and leave-one-out plots of the Mendelian randomization analyses between genetically predicted fetuin-A and myocardial infarction within the UK Biobank using the genetic variants used in the study from Fisher et al. A) Scatter plot: the variants’ effect size and standard error on fetuin-A are displayed on the X-axis, the variants’ effect size and standard error on the outcomes are displayed on the Y-axis. The blue line is the regression line of the inverse variance weighted multiplicative random effects meta-analysis, the green line of the MR contamination mixture model, the red line of the MR-Egger analysis, the orange line of the MR Lasso method and the purple line of the weighted median method. Top right: forest plot. The effect size in beta and SE are displayed on the X-axis, the genetic variants or statistic test are displayed on the X-axis. Wald estimates are displayed in black, pooled effect estimates in red. Bottom left: funnel plot. The beta of the Wald estimate per SNP is displayed on the X-axis, the Y-axis displays 1/standard error of the Wald estimate. The light blue line demonstrates the results of the inverse variance weighted analysis, dark blue the results of the MR-Egger analysis. Bottom right: leave-one-out plot. The effect size in beta and SE are displayed on the X-axis, the genetic variants left out or the pooled inverse variance weighted effect estimate of all included SNPs are displayed on the Y-axis. SNP denotes single nucleotide polymorphism, MR denotes Mendelian randomization.*

C) Fetuin-A – any stroke

**
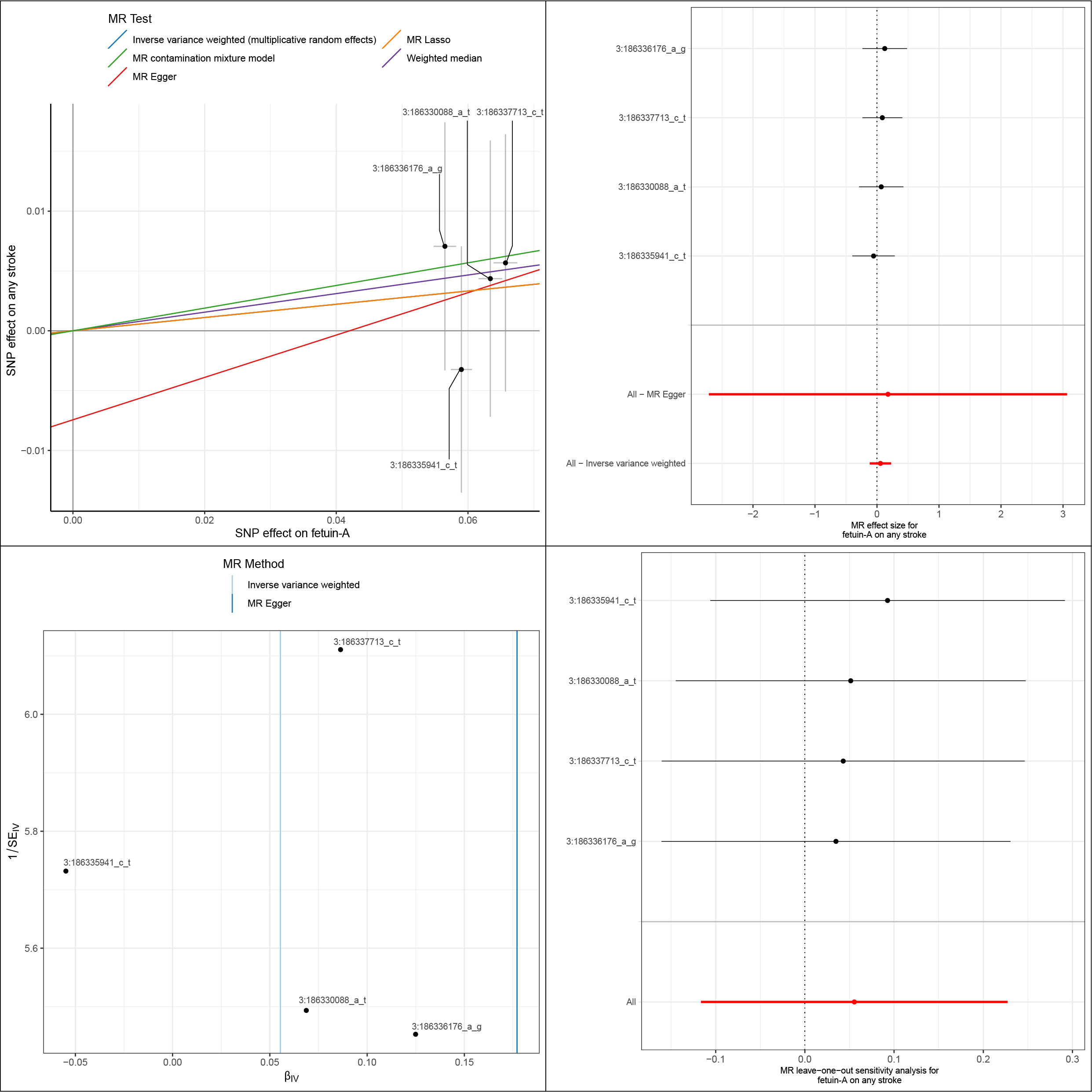
**

*Scatterplots, forest plots, funnel plots, and leave-one-out plots of the Mendelian randomization analyses between genetically predicted fetuin-A and any stroke within the UK Biobank using the genetic variants used in the study from Fisher et al. A) Scatter plot: the variants’ effect size and standard error on fetuin-A are displayed on the X-axis, the variants’ effect size and standard error on the outcomes are displayed on the Y-axis. The blue line is the regression line of the inverse variance weighted multiplicative random effects meta-analysis, the green line of the MR contamination mixture model, the red line of the MR-Egger analysis, the orange line of the MR Lasso method and the purple line of the weighted median method. Top right: forest plot. The effect size in beta and SE are displayed on the X-axis, the genetic variants or statistic test are displayed on the X-axis. Wald estimates are displayed in black, pooled effect estimates in red. Bottom left: funnel plot. The beta of the Wald estimate per SNP is displayed on the X-axis, the Y-axis displays 1/standard error of the Wald estimate. The light blue line demonstrates the results of the inverse variance weighted analysis, dark blue the results of the MR-Egger analysis. Bottom right: leave-one-out plot. The effect size in beta and SE are displayed on the X-axis, the genetic variants left out or the pooled inverse variance weighted effect estimate of all included SNPs are displayed on the Y-axis. SNP denotes single nucleotide polymorphism, MR denotes Mendelian randomization.*

D) Fetuin-A – any ischemic stroke

*
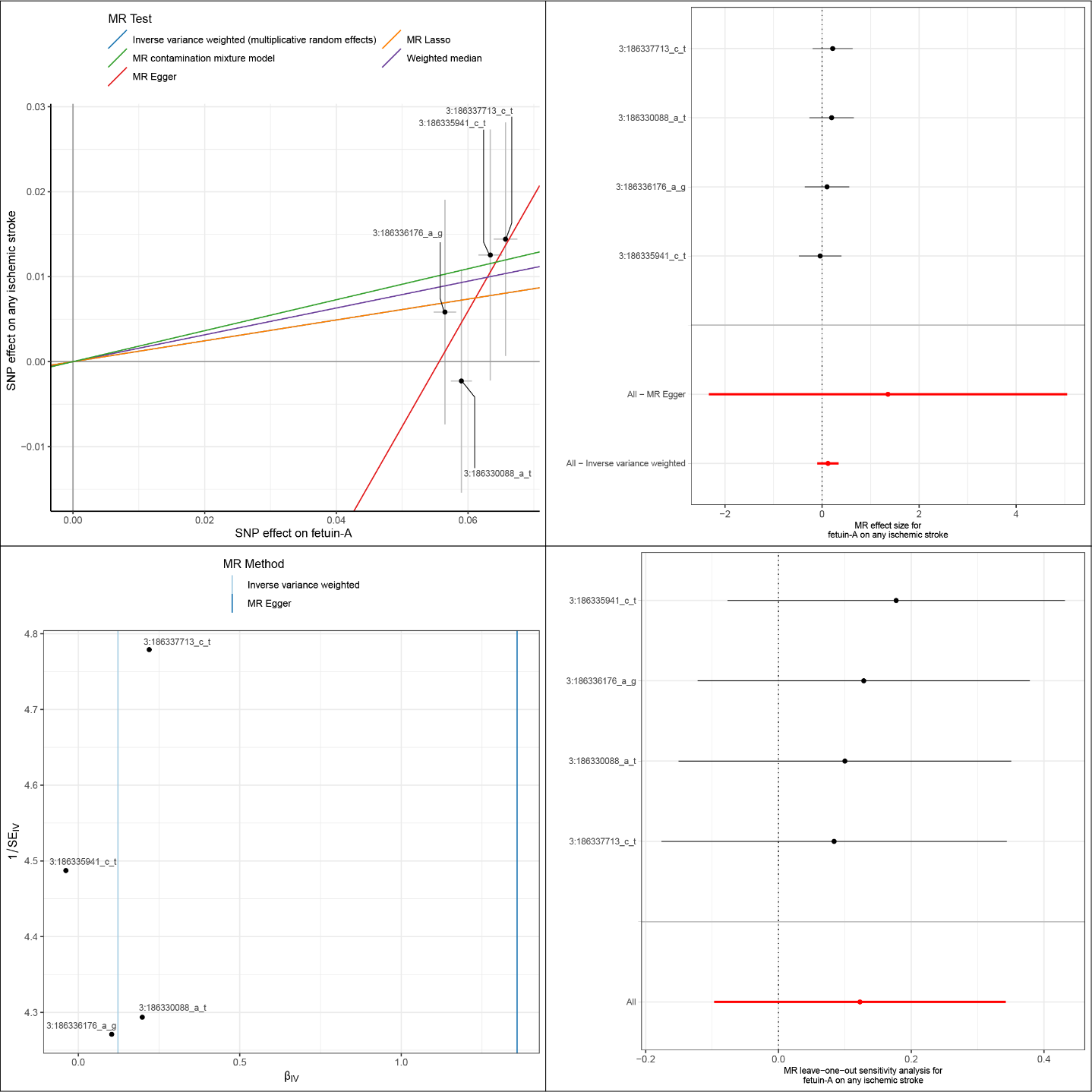
*

*Scatterplots, forest plots, funnel plots, and leave-one-out plots of the Mendelian randomization analyses between genetically predicted fetuin-A and any ischemic stroke within the UK Biobank using the genetic variants used in the study from Fisher et al. A) Scatter plot: the variants’ effect size and standard error on fetuin-A are displayed on the X-axis, the variants’ effect size and standard error on the outcomes are displayed on the Y-axis. The blue line is the regression line of the inverse variance weighted multiplicative random effects meta-analysis, the green line of the MR contamination mixture model, the red line of the MR-Egger analysis, the orange line of the MR Lasso method and the purple line of the weighted median method. Top right: forest plot. The effect size in beta and SE are displayed on the X-axis, the genetic variants or statistic test are displayed on the X-axis. Wald estimates are displayed in black, pooled effect estimates in red. Bottom left: funnel plot. The beta of the Wald estimate per SNP is displayed on the X-axis, the Y-axis displays 1/standard error of the Wald estimate. The light blue line demonstrates the results of the inverse variance weighted analysis, dark blue the results of the MR-Egger analysis. Bottom right: leave-one-out plot. The effect size in beta and SE are displayed on the X-axis, the genetic variants left out or the pooled inverse variance weighted effect estimate of all included SNPs are displayed on the Y-axis. SNP denotes single nucleotide polymorphism, MR denotes Mendelian randomization.*

E) Fetuin-A – type 2 diabetes

*
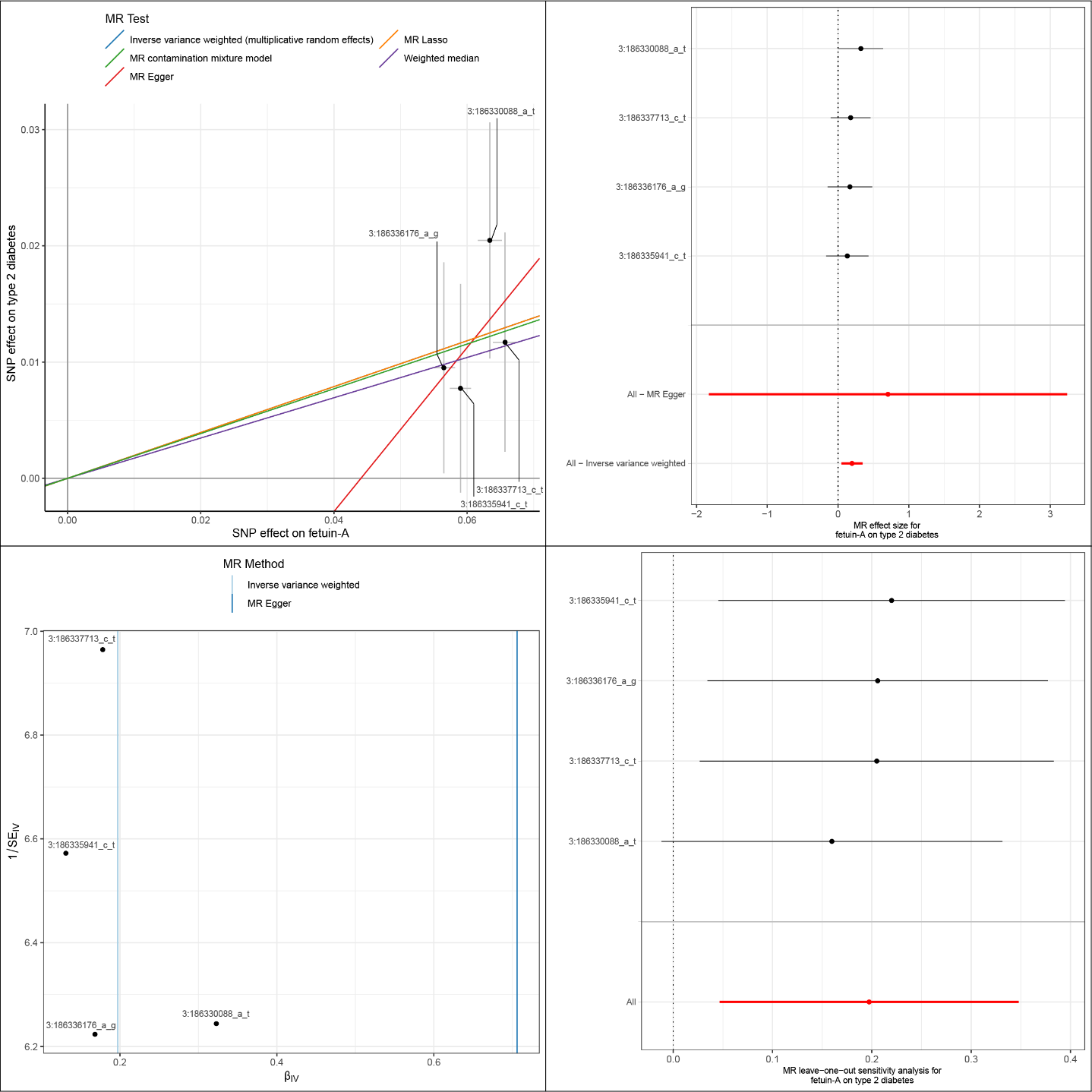
*

*Scatterplots, forest plots, funnel plots, and leave-one-out plots of the Mendelian randomization analyses between genetically predicted fetuin-A and type 2 diabetes within the UK Biobank using the genetic variants used in the study from Fisher et al. A) Scatter plot: the variants’ effect size and standard error on fetuin-A are displayed on the X-axis, the variants’ effect size and standard error on the outcomes are displayed on the Y-axis. The blue line is the regression line of the inverse variance weighted multiplicative random effects meta-analysis, the green line of the MR contamination mixture model, the red line of the MR-Egger analysis, the orange line of the MR Lasso method and the purple line of the weighted median method. Top right: forest plot. The effect size in beta and SE are displayed on the X-axis, the genetic variants or statistic test are displayed on the X-axis. Wald estimates are displayed in black, pooled effect estimates in red. Bottom left: funnel plot. The beta of the Wald estimate per SNP is displayed on the X-axis, the Y-axis displays 1/standard error of the Wald estimate. The light blue line demonstrates the results of the inverse variance weighted analysis, dark blue the results of the MR-Egger analysis. Bottom right: leave-one-out plot. The effect size in beta and SE are displayed on the X-axis, the genetic variants left out or the pooled inverse variance weighted effect estimate of all included SNPs are displayed on the Y-axis. SNP denotes single nucleotide polymorphism, MR denotes Mendelian randomization.*

**Supplementary Figure 5:** Scatterplots, forest plots, funnel plots, and leave-one-out plots of the Mendelian randomization analyses of genetically predicted fetuin-A with cardiovascular diseases and type 2 diabetes, using all independent genetic variants used in the study of Kröger *et al*.

A) Fetuin-A – coronary artery disease

**
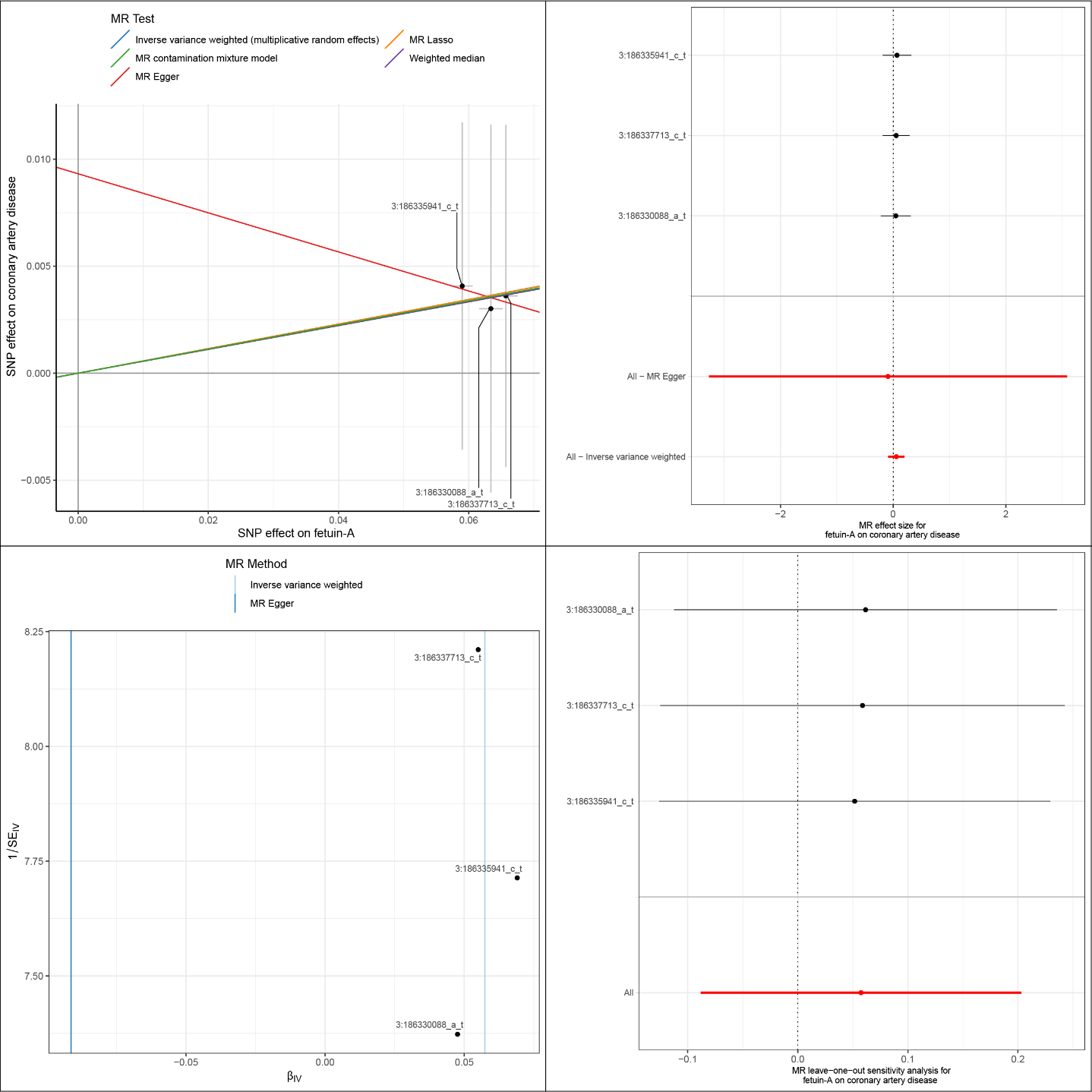
**

*Scatterplots, forest plots, funnel plots, and leave-one-out plots of the Mendelian randomization analyses between genetically predicted fetuin-A and coronary artery disease within the UK Biobank using the genetic variants used in the study from Kröger et al. Top left: scatter plot. The variants’ effect size and standard error on fetuin-A are displayed on the X-axis, the variants’ effect size and standard error on the outcomes are displayed on the Y-axis. The blue line is the regression line of the inverse variance weighted multiplicative random effects meta-analysis, the green line of the MR contamination mixture model, the red line of the MR-Egger analysis, the orange line of the MR Lasso method and the purple line of the weighted median method. Top right: forest plot. The effect size in beta and SE are displayed on the X-axis, the genetic variants or statistic test are displayed on the X-axis. Wald estimates are displayed in black, pooled effect estimates in red. Bottom left: funnel plot. The beta of the Wald estimate per SNP is displayed on the X-axis, the Y-axis displays 1/standard error of the Wald estimate. The light blue line demonstrates the results of the inverse variance weighted analysis, dark blue the results of the MR-Egger analysis. Bottom right: leave-one-out plot. The effect size in beta and SE are displayed on the X-axis, the genetic variants left out or the pooled inverse variance weighted effect estimate of all included SNPs are displayed on the Y-axis. SNP denotes single nucleotide polymorphism, MR denotes Mendelian randomization.*

B) Fetuin-A – myocardial infarction

**
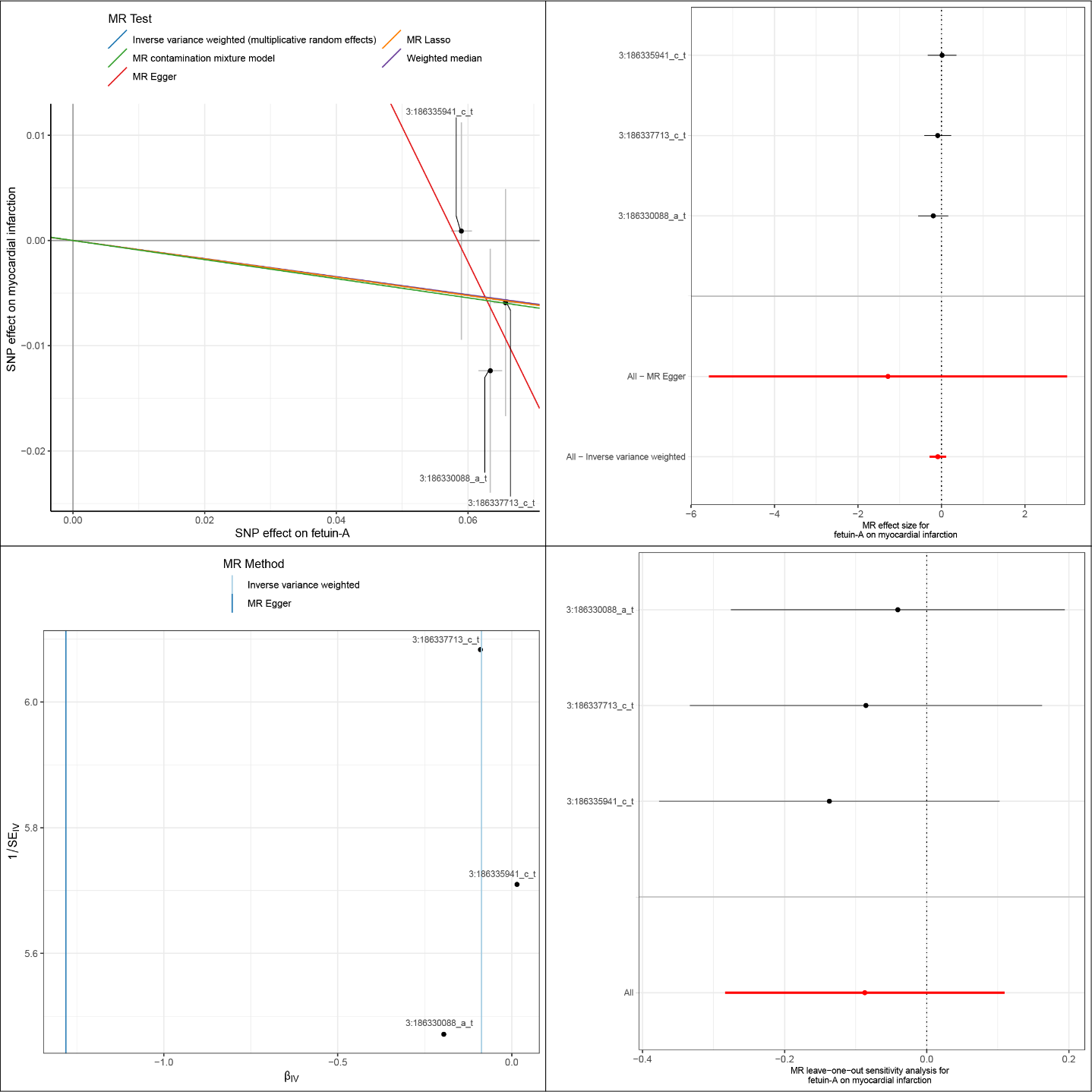
**

*Scatterplots, forest plots, funnel plots, and leave-one-out plots of the Mendelian randomization analyses between genetically predicted fetuin-A and myocardial infarction within the UK Biobank using the genetic variants used in the study from Kröger et al. Top left: scatter plot. The variants’ effect size and standard error on fetuin-A are displayed on the X-axis, the variants’ effect size and standard error on the outcomes are displayed on the Y-axis. The blue line is the regression line of the inverse variance weighted multiplicative random effects meta-analysis, the green line of the MR contamination mixture model, the red line of the MR-Egger analysis, the orange line of the MR Lasso method and the purple line of the weighted median method. Top right: forest plot. The effect size in beta and SE are displayed on the X-axis, the genetic variants or statistic test are displayed on the X-axis. Wald estimates are displayed in black, pooled effect estimates in red. Bottom left: funnel plot. The beta of the Wald estimate per SNP is displayed on the X-axis, the Y-axis displays 1/standard error of the Wald estimate. The light blue line demonstrates the results of the inverse variance weighted analysis, dark blue the results of the MR-Egger analysis. Bottom right: leave-one-out plot. The effect size in beta and SE are displayed on the X-axis, the genetic variants left out or the pooled inverse variance weighted effect estimate of all included SNPs are displayed on the Y-axis. SNP denotes single nucleotide polymorphism, MR denotes Mendelian randomization.*

C) Fetuin-A – any stroke

*
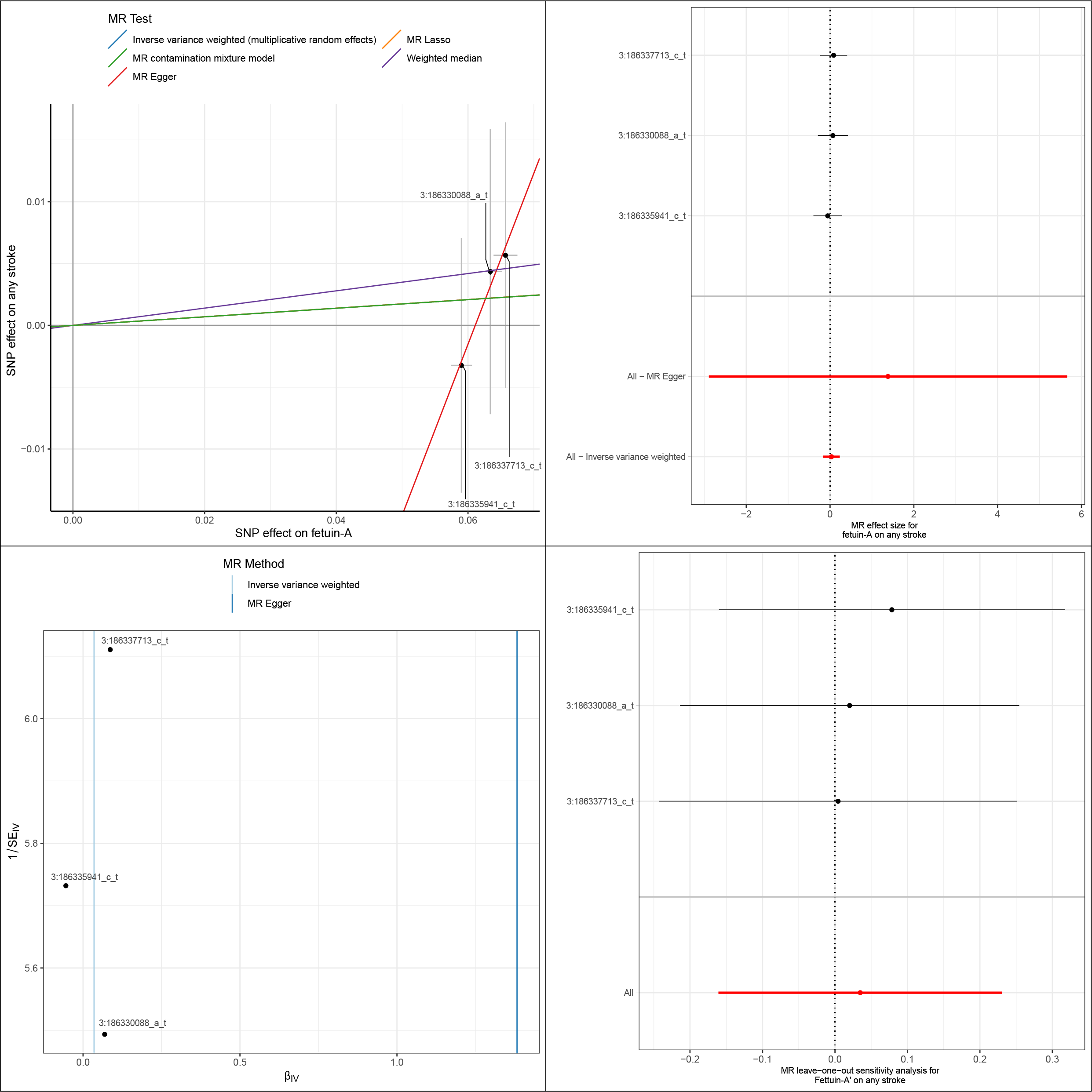
*

*Scatterplots, forest plots, funnel plots, and leave-one-out plots of the Mendelian randomization analyses between genetically predicted fetuin-A and any stroke within the UK Biobank using the genetic variants used in the study from Kröger et al. Top left: scatter plot. The variants’ effect size and standard error on fetuin-A are displayed on the X-axis, the variants’ effect size and standard error on the outcomes are displayed on the Y-axis. The blue line is the regression line of the inverse variance weighted multiplicative random effects meta-analysis, the green line of the MR contamination mixture model, the red line of the MR-Egger analysis, the orange line of the MR Lasso method and the purple line of the weighted median method. Top right: forest plot. The effect size in beta and SE are displayed on the X-axis, the genetic variants or statistic test are displayed on the X-axis. Wald estimates are displayed in black, pooled effect estimates in red. Bottom left: funnel plot. The beta of the Wald estimate per SNP is displayed on the X-axis, the Y-axis displays 1/standard error of the Wald estimate. The light blue line demonstrates the results of the inverse variance weighted analysis, dark blue the results of the MR-Egger analysis. Bottom right: leave-one-out plot. The effect size in beta and SE are displayed on the X-axis, the genetic variants left out or the pooled inverse variance weighted effect estimate of all included SNPs are displayed on the Y-axis. SNP denotes single nucleotide polymorphism, MR denotes Mendelian randomization.*

D) Fetuin-A – any ischemic stroke

*
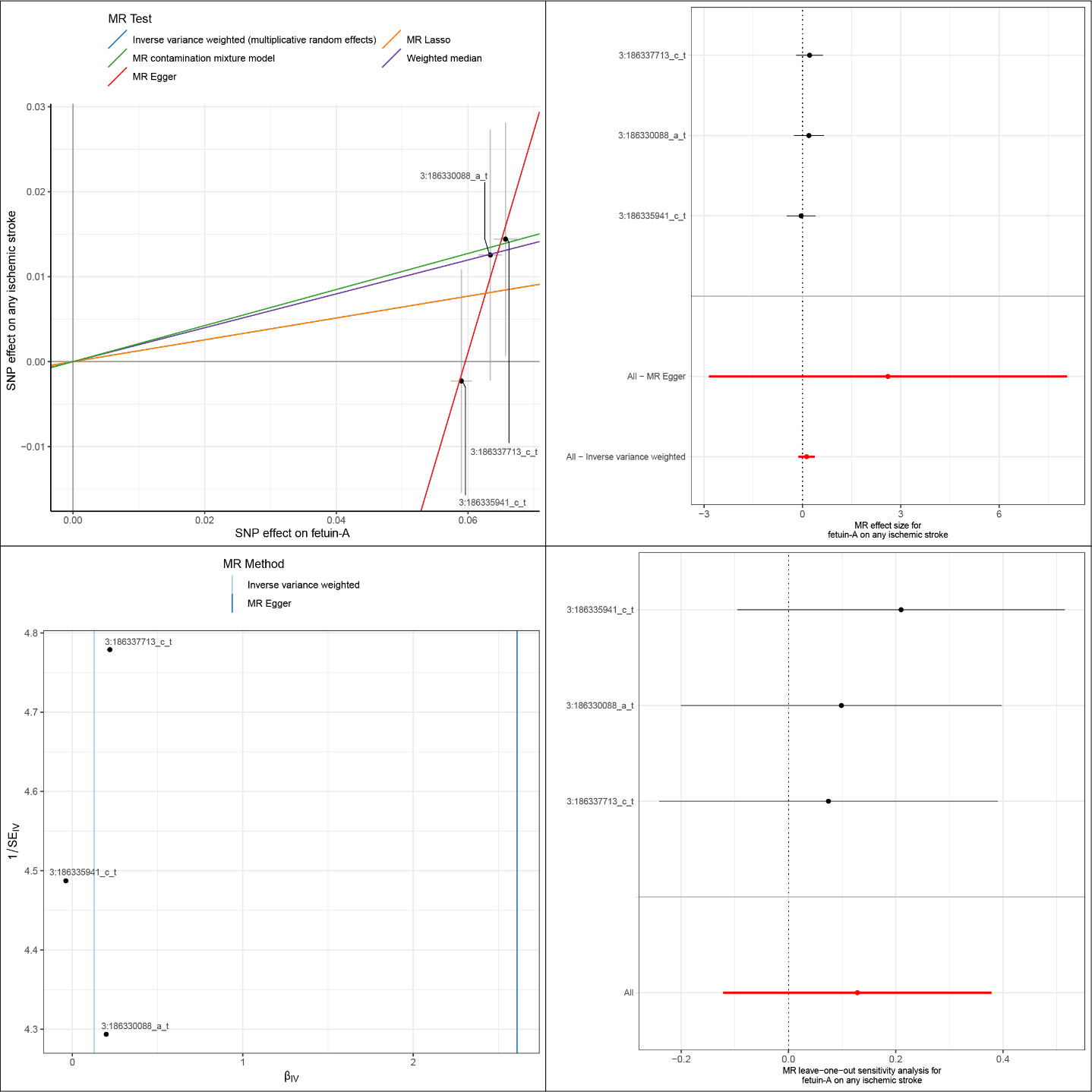
*

*Scatterplots, forest plots, funnel plots, and leave-one-out plots of the Mendelian randomization analyses between genetically predicted fetuin-A and any ischemic stroke within the UK Biobank using the genetic variants used in the study from Kröger et al. Top left: scatter plot. The variants’ effect size and standard error on fetuin-A are displayed on the X-axis, the variants’ effect size and standard error on the outcomes are displayed on the Y-axis. The blue line is the regression line of the inverse variance weighted multiplicative random effects meta-analysis, the green line of the MR contamination mixture model, the red line of the MR-Egger analysis, the orange line of the MR Lasso method and the purple line of the weighted median method. Top right: forest plot. The effect size in beta and SE are displayed on the X-axis, the genetic variants or statistic test are displayed on the X-axis. Wald estimates are displayed in black, pooled effect estimates in red. Bottom left: funnel plot. The beta of the Wald estimate per SNP is displayed on the X-axis, the Y-axis displays 1/standard error of the Wald estimate. The light blue line demonstrates the results of the inverse variance weighted analysis, dark blue the results of the MR-Egger analysis. Bottom right: leave-one-out plot. The effect size in beta and SE are displayed on the X-axis, the genetic variants left out or the pooled inverse variance weighted effect estimate of all included SNPs are displayed on the Y-axis. SNP denotes single nucleotide polymorphism, MR denotes Mendelian randomization.*

E) Fetuin-A – type 2 diabetes

*
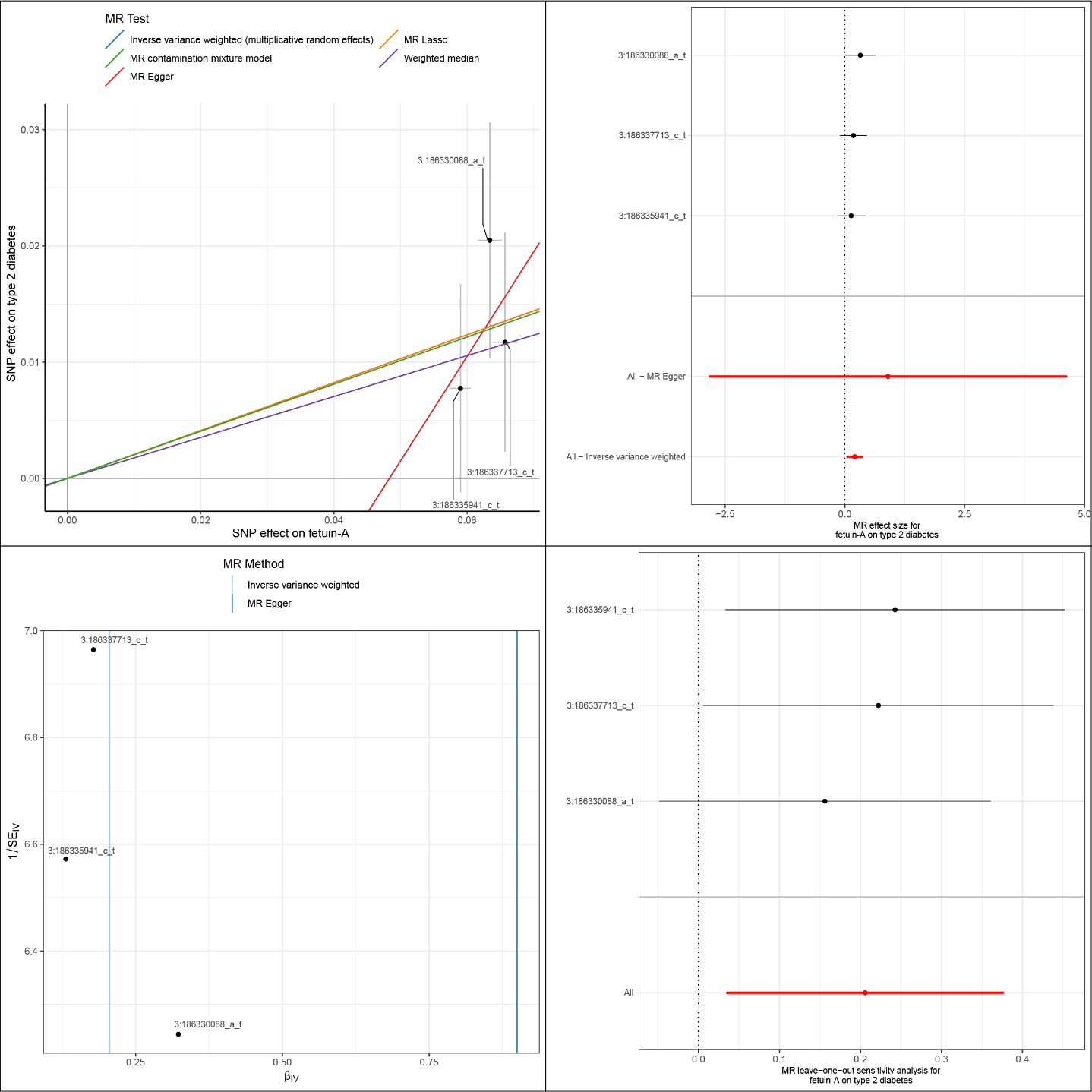
*

*Scatterplots, forest plots, funnel plots, and leave-one-out plots of the Mendelian randomization analyses between genetically predicted fetuin-A and any type 2 diabetes within the UK Biobank using the genetic variants used in the study from Kröger et al. Top left: scatter plot. The variants’ effect size and standard error on fetuin-A are displayed on the X-axis, the variants’ effect size and standard error on the outcomes are displayed on the Y-axis. The blue line is the regression line of the inverse variance weighted multiplicative random effects meta-analysis, the green line of the MR contamination mixture model, the red line of the MR-Egger analysis, the orange line of the MR Lasso method and the purple line of the weighted median method. Top right: forest plot. The effect size in beta and SE are displayed on the X-axis, the genetic variants or statistic test are displayed on the X-axis. Wald estimates are displayed in black, pooled effect estimates in red. Bottom left: funnel plot. The beta of the Wald estimate per SNP is displayed on the X-axis, the Y-axis displays 1/standard error of the Wald estimate. The light blue line demonstrates the results of the inverse variance weighted analysis, dark blue the results of the MR-Egger analysis. Bottom right: leave-one-out plot. The effect size in beta and SE are displayed on the X-axis, the genetic variants left out or the pooled inverse variance weighted effect estimate of all included SNPs are displayed on the Y-axis. SNP denotes single nucleotide polymorphism, MR denotes Mendelian randomization.*

**Supplementary Figure 6: Directed acyclic graph of the core assumptions made in Mendelian randomization analyses.**


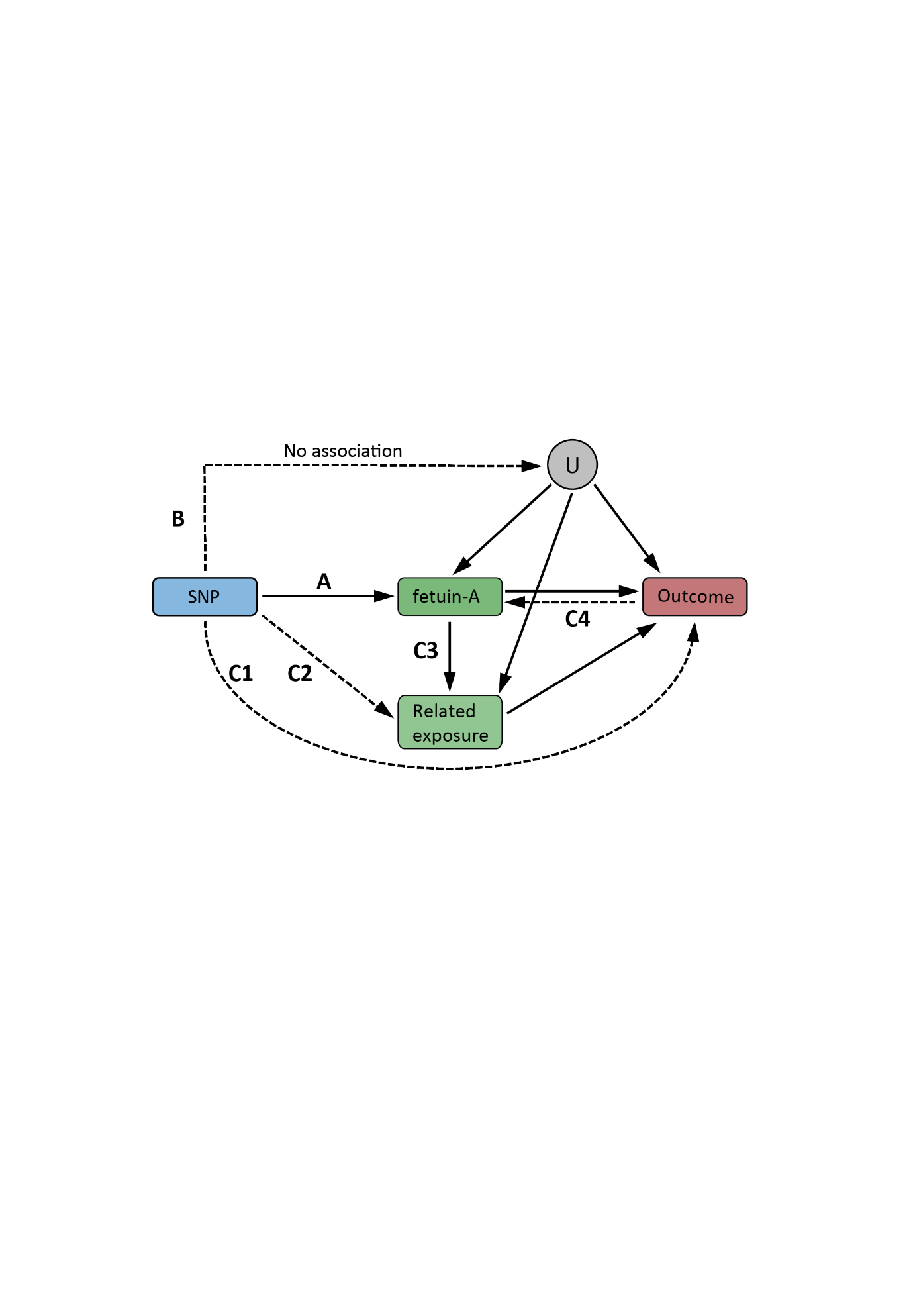
*Directed acyclic graph of the core assumptions made in Mendelian randomization analyses. Shown are the effects of a single nucleotide polymorphism (SNP) as instrumental variable (blue), the exposure (shown in green, for example fetuin-A), a related exposure (shown in green), the outcome (shown in red, for example coronary artery disease), and unobserved confounders (U, shown in grey). Solid lines indicate potential associations, dashed lines indicate that there should be no association for the MR assumptions to be fulfilled. The letters correspond to the three conditions that have to be satisfied for correctly inferring a potential causal relationship, including* ***A)*** *a strong and reliable association between the genetic variant and fetuin-A,* ***B)*** *independence of unobserved confounding through fetuin-A, and* ***C)*** *the association between genetically predicted fetuin-A and the outcomes is caused through fetuin-A.^1^. Condition* ***C)*** *is divided into four subparts.* ***C1)*** *represents unbalanced horizontal pleiotropy, a scenario in which the effect of the genetic variant on the exposure is zero, but still affects the outcome.* ***C2)*** *represents horizontal pleiotropic effects through another (related) exposure. The genetic variant affects the exposure (i.e., fetuin-A) and a (related) exposure. Both the exposure and related exposure in turn affect the outcome independently.* ***C3)*** *represents vertical pleiotropic effects through another (related) exposure. In this scenario, a genetic variant exerts an effect on the outcome through the exposure and the related exposure, but not this effect is not caused by the effect of the genetic variant on the related exposure.* ***C4)*** *represents reversed causation. A genetic variant can affect the exposure through the outcome instead of the outcome through the exposure. The letters (A, B and C) correspond with the Mendelian randomization assumptions described in the main text and with the letters in* ***Supplementary Figure 7****, in which several methods to assess and account for potential violations of Mendelian randomization assumptions can be found.*

**Supplementary Figure 7. Theoretical overview of Mendelian randomization analyses.**

*
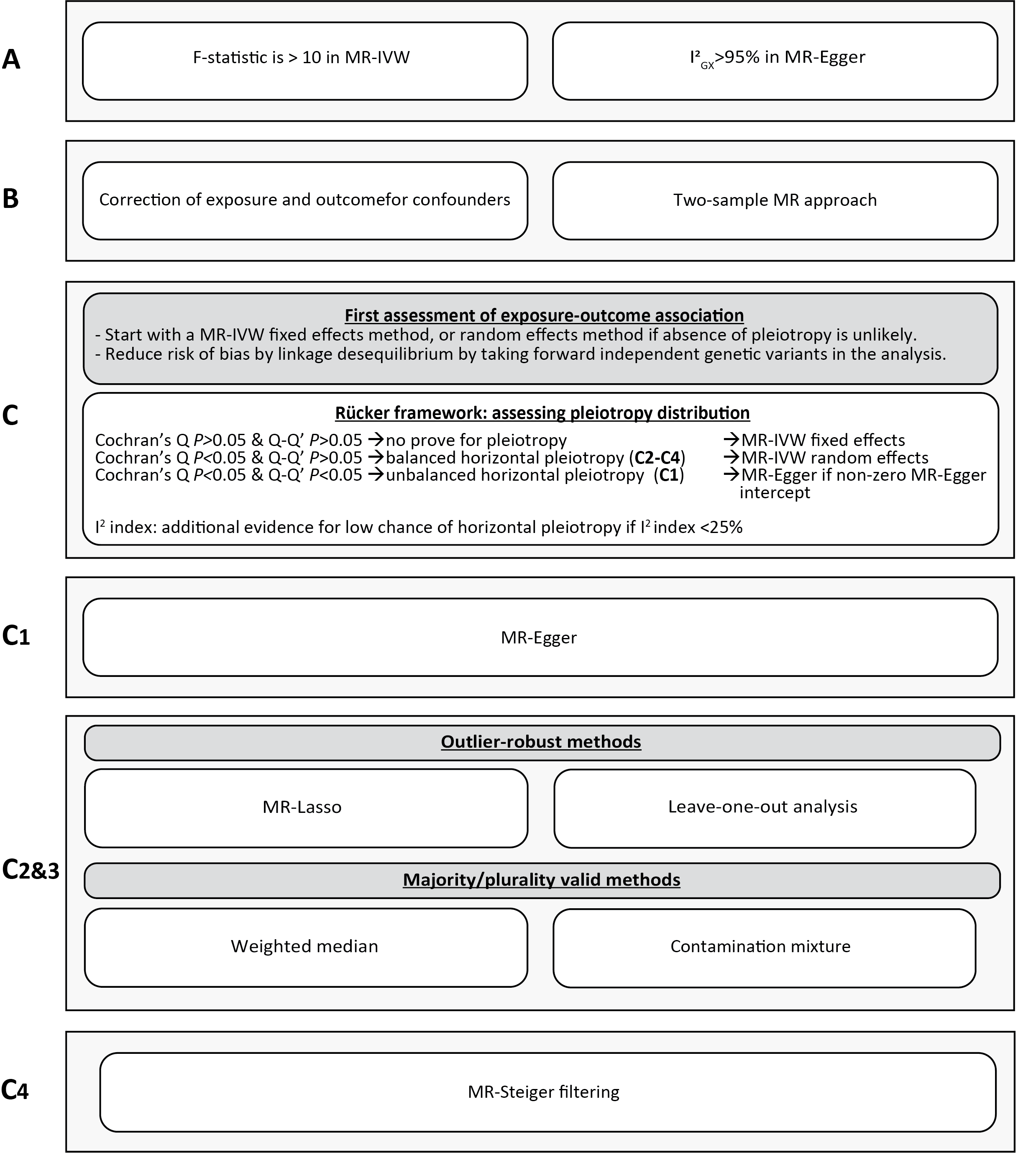
*

*The letters correspond to the three conditions that have to be satisfied for correctly inferring a potential causal relationship, including* ***A)*** *a strong and reliable association between the genetic variant and fetuin-A,* ***B)*** *independence of unobserved confounding through fetuin-A, and* ***C)*** *the association between genetically predicted fetuin-A and the outcomes is caused through fetuin-A.^1^*
***Assumption A)****, the variant is predictive of the exposure, can be assessed using the F-statistics when using a two-sample univariable MR-inverse-variance weighted (IVW) analysis^2^. In case a univariable MR-Egger approach is used, weak instrument bias can be assessed using the I^2^_GX_ index^3^.*
***Assumption B)****, the variant is independent of any confounding factors of the exposure-outcome association, cannot be proven. However, several precautions can be undertaken to make it less likely. Confounding is, in theory, limited by the second law of Mendel, which states that genetic variants for different traits are inherited independently^1^. In addition, both the regression analyses in both the exposure and outcome cohorts in the current study were corrected for the most important confounders, including age, sex, and population stratification. The exposure and outcome cohorts were corrected for the same covariates. This study adopted a two-sample Mendelian randomization approach, in which independent exposure and outcome cohorts were used. The use of a two-sample Mendelian randomization approach reduces the risk that potential weak-instrument bias increases type 1 error rates through reintroduction of confounding*^4^*.*
***Assumption*** ***C)****, the genetic variant affects the outcome only through the exposure, is also technically impossible to prove. However, it can be made less likely or possibly be taken into account by performing several sensitivity analyses. The first assessment of a linear exposure-outcome association in the current study was performed using an univariable inverse variance weighted (MR-IVW)*^5^ *and MR-Egger analysis*^6^*. The Rücker framework was used to differentiate between different types of heterogeneity and thus potential pleiotropy*^7^*.The Cochran’s Q provides information on the heterogeneity within the MR-IVW estimate. A Cochran’s Q P-value of <0.05 is considered proof of heterogeneity and therefore indicative of at least potential horizontal pleiotropy within the MR-IVW estimate*^7^*. This conclusion is further supported by an I^2^ index > 25%*^8^*. In the scenario of balanced horizontal pleiotropy (****C2&3****), one can obtain a consistent estimate of the exposure-outcome association through the use of an MR-IVW multiplicative random-effects model*^5,7^ *The Rücker framework also requires calculation of the heterogeneity within the MR-Egger regression (Rucker’s Q) and calculation of the difference between heterogeneity within the MR-IVW and MR-Egger effect estimates (Q-Q')*^7^*. The framework indicates potential unbalanced horizontal pleiotropy if the results show a significant Q-Q' (P-value<0.05) in combination with a significant non-zero intercept of the MR-Egger regression (P-value <0.05)*^7^*.*
*In* ***scenario*** ***C1*** *(unbalanced horizontal pleiotropy), the genetic variant exerts an effect on the outcome when its effect on the exposure is zero. In this scenario, a consistent and potential causal estimate can be obtained through an MR-Egger model. The MR-Egger can provide causal estimates if SNPs exert unbalanced horizontal pleiotropy, under the assumption that Instrument Strength Independent of Direct Effect (InSIDE) assumption holds*^6^*.*
*In* ***scenario*** ***C2&3*** *(balanced pleiotropic effects), there is an effect of a related exposure on the outcome. This can be either through the effect of the genetic variant directly on the related exposure (****C2,*** *horizontal pleiotropy), or through the effect on of the exposure on the related exposure not caused by the effect of the genetic variant on the related exposure (****C3,*** *vertical pleiotropy). Balanced horizontal pleiotropic effects can be driven by several or a larger group of outliers that distort the exposure-outcome association. In the scenario that a small proportion of the genetic variants is invalid and show heterogeneous ratio estimates, a consistent estimate for genetic variants not identified as outliers can obtained under the same assumptions as the MR-IVW method using the MR-Lasso*^9^ *or leave-one-out analysis*^10^*. A disadvantage of leave-one-out analysis compared to newer methods such as MR-lasso is that leave-one-out analysis forces the exclusion of genetic variants and that the percentage of removed genetic variants depends largely on the number of independent variants included in the study. For example, compared to performing a leave-one-out analysis with for example 500 genetic variants, leave-one-out analysis with only three genetic variants reduces the total amount of instruments with a significant amount. Other methods allow for a generally consistent effect estimate in the scenario that a large proportion of the genetic variants is invalid and show heterogeneous ratio estimates. A consistent estimate for half of the largest subset of valid variants can be obtained using the weighed median*^11^ *and MR contamination mixture methods*^12^*.*
*In* ***scenario C4*** *(reversed causation), a genetic variant affects the exposure through the outcome instead of the other way around. This can be assessed by performing MR-Steiger filtering, which might be used to remove genetic variants from the MR analysis if their association is stronger with the outcome than the exposure*^13^*.*
***Assumption C)*** *can also be potentially violated by linkage disequilibrium*^14^*. Linkage disequilibrium is a correlation between allelic states at different loci on a part of the same chromosome when assessed within a certain population*^14^*. Through this mechanism, the tagged genetic variant can potentially be in linkage disequilibrium with another variant that affects the outcome via another pathway than through the exposure*^14^*. We therefore used a stringent clumping procedure to select independent genetic variants for the Mendelian randomization analysis.*

**Supplementary Table 1:** Mendelian randomization analysis of fetuin-A on type 2 diabetes mellitus, using the independent genetic variants and effect sizes used in a previous study of Kröger *et al.*

| **Method** | **Nsnp** | **Beta** | **Se** | ***P*-value** | **OR** | **95% CI min** | **95% CI plus** | **Noutcome** | **Ncontrol** |
| --- | --- | --- | --- | --- | --- | --- | --- | --- | --- |
| Inverse variance weighted (fixed effects) | 3 | 0.165 | 0.128 | 0.199 | 1.179 | 0.917 | 1.515 | 28333 | 374956 |
| Inverse variance weighted (multiplicative random effects) | 3 | 0.165 | 0.196 | 0.402 | 1.179 | 0.802 | 1.732 | 28333 | 374956 |
| MR Egger | 3 | 0.932 | 12.141 | 0.951 | 2.541 | 0.000 | 54934464025.492 | 28333 | 374956 |
| Weighted median | 3 | 0.209 | 0.175 | 0.232 | 1.232 | 0.875 | 1.736 | 28333 | 374956 |
| MR Lasso | 3 | 0.165 | 0.196 | 0.402 | 1.179 | 0.802 | 1.732 | 28333 | 374956 |
| MR contamination mixture model | 3 | 0.362 | 0.244 | 0.137 | 1.437 | 0.891 | 2.317 | 28333 | 374956 |

*We did not find evidence for horizontal pleiotropy in the current analyses (I2 index = 57.4%,95%CI= 0.0-87.94; Cochran’s Q = 4.7, df = 2; P = 0.10; Q-Q’ = 0.02, df = 1, P = 0.89; MR-Egger intercept -0.0033 ± 0.519, P = 0.96). There was no evidence for weak instrument bias in the MR-Egger regression (I^2^_GX_ = 0.99)*

**Supplementary refences**

1. Davey Smith, G. & Hemani, G. Mendelian randomization: genetic anchors for causal inference in epidemiological studies. *Hum. Mol. Genet.* 23, R89–R98 (2014).

2. Palmer, T. M., Lawlor, D. A., Harbord, R. M., *et al.* Using multiple genetic variants as instrumental variables for modifiable risk factors. in *Statistical Methods in Medical Research* vol. 21 223–242 (2012).

3. Bowden, J., Del Greco M., F., Minelli, C., *et al.* Assessing the suitability of summary data for two-sample Mendelian randomization analyses using MR-Egger regression: the role of the I2 statistic. *Int. J. Epidemiol.* 45, dyw220 (2016).

4. Burgess, S. & Thompson, S. G. Avoiding bias from weak instruments in Mendelian randomization studies. *Int. J. Epidemiol.* 40, 755–764 (2011).

5. Burgess, S., Butterworth, A. & Thompson, S. G. Mendelian randomization analysis with multiple genetic variants using summarized data. *Genet. Epidemiol.* 37, 658–665 (2013).

6. Bowden, J., Davey Smith, G. & Burgess, S. Mendelian randomization with invalid instruments: effect estimation and bias detection through Egger regression. *Int. J. Epidemiol.* 44, 512–525 (2015).

7. Bowden, J., Del Greco M, F., Minelli, C., *et al.* A framework for the investigation of pleiotropy in two-sample summary data Mendelian randomization. *Stat. Med.* 36, 1783–1802 (2017).

8. Greco M, F. Del, Minelli, C., Sheehan, N. A. & Thompson, J. R. Detecting pleiotropy in Mendelian randomisation studies with summary data and a continuous outcome. *Stat. Med.* 34, 2926–2940 (2015).

9. Rees, J. M. B., Wood, A. M., Dudbridge, F. & Burgess, S. Robust methods in Mendelian randomization via penalization of heterogeneous causal estimates. *PLoS One* 14, e0222362 (2019).

10. Corbin, L. J., Richmond, R. C., Wade, K. H., *et al.* Body mass index as a modifiable risk factor for type 2 diabetes: Refining and understanding causal estimates using Mendelian randomisation. *Diabetes* 65, 3002 (2016).

11. Bowden, J., Davey Smith, G., Haycock, P. C. & Burgess, S. Consistent Estimation in Mendelian Randomization with Some Invalid Instruments Using a Weighted Median Estimator. *Genet. Epidemiol.* 40, 304–314 (2016).

12. Burgess, S., Foley, C. N., Allara, E., Staley, J. R. & Howson, J. M. M. A robust and efficient method for Mendelian randomization with hundreds of genetic variants. *Nat. Commun.* 11, 1–11 (2020).

13. Hemani, G., Tilling, K. & Davey Smith, G. Orienting the causal relationship between imprecisely measured traits using GWAS summary data. *PLoS Genet.* 13, e1007081 (2017).

14. Smith, G. D. & Ebrahim, S. ‘Mendelian randomization’: Can genetic epidemiology contribute to understanding environmental determinants of disease? *International Journal of Epidemiology* vol. 32 1–22 (2003).
